# Dynamic redox–promoted iron and nutrient cycling drove graptolite evolution across the Ordovician-Silurian transition

**DOI:** 10.1126/sciadv.aea1423

**Published:** 2026-01-21

**Authors:** Zhen Qiu, Caineng Zou, Jiaqiang Zhang, Aiguo Dong, Weiliang Kong, Yijun Xiong, Paul B. Wignall, Ming Li, Zaicong Wang, Xiangkun Zhu, Simon W. Poulton

**Affiliations:** ^1^ Research Institute of Petroleum Exploration & Development, China National Petroleum Corporation, Beijing, China.; ^2^ School of Geoscience and Technology, Southwest Petroleum University, Chengdu, China.; ^3^ School of Earth and Environment, University of Leeds, Leeds LS2 9JT, UK.; ^4^ Institute of Earth Sciences, China University of Geosciences (Beijing), Beijing, China.; ^5^ State Key Laboratory of Geological Processes and Mineral Resources, School of Earth Sciences, China University of Geosciences (Wuhan), Wuhan, China.; ^6^ MNR Key Laboratory of Isotope Geology, MNR Key Laboratory of Deep-Earth Dynamics, Institute of Geology, Chinese Academy of Geological Sciences, Beijing, China.

## Abstract

Graptolites were abundant and cosmopolitan zooplankton in Early Paleozoic oceans, but a prominent change in species occurred across the Late Ordovician mass extinction. We use ocean redox, iron isotope (δ^56^Fe), and phosphorus phase partitioning records from shelf and deep-ocean settings to evaluate the drivers behind this major reshaping of the pelagic marine ecosystem. A marked decrease in mesopelagic graptolites coincided with a stepwise negative δ^56^Fe shift on the shelf, driven by partial seawater Fe drawdown resulting from episodic intensification of mid-depth euxinia. Subsequently, a positive δ^56^Fe shift in both deep-ocean and shelf sediments reflects extensive seawater Fe removal during the development of more widespread euxinia. This led to enhanced sedimentary phosphorus recycling from sediments, which ultimately fueled the radiation of epipelagic graptolites. Thus, wide-scale changes in Fe cycling, linking the global oceanic redox state to phosphorus cycling, were ultimately responsible for the initial demise and subsequent radiation of select graptolite species.

## Teaser

Fe-driven nutrient cycling was responsible for graptolite evolution across the Ordovician-Silurian transition.

## INTRODUCTION

Dissolved oxygen (O_2_) is critical for the evolution and survival of marine life. The appearance of the first animals in the Ediacaran ocean (*1*) indicates that O_2_ levels were sufficient to support their specific metabolisms at this time (*2*, *3*). However, there is increasing evidence that shallower waters were commonly characterized by relatively low O_2_ levels (i.e., dysoxic conditions) in the Early Paleozoic (*4*–*6*), while mid-to-deep oceanic environments were often anoxic, before the more widespread and persistent development of fully oxygenated deep oceans in the Devonian, about 400 million years ago (*7*–*9*).

Although Early Paleozoic marine animals appear to have been adapted to low-O_2_ oceanic conditions (*4*, *5*), the dynamic intensification and expansion of ocean anoxia imposed severe stress on marine life, resulting in catastrophic biological crises such as the Late Ordovician mass extinction (LOME) (*10*–*13*). As the first of the “Big Five” mass extinctions of the Phanerozoic, the LOME is estimated to have wiped out nearly 85% of marine species (*14*, *15*). This extinction event profoundly reshaped the structure and evolutionary trajectory of macroplanktonic communities, including graptolites (*16*).

Graptolites were abundant and cosmopolitan zooplankton in Early Paleozoic oceans, with two major clades: the Diplograptina and Neograptina (*16*, *17*). Before the LOME, the dominant Diplograptina mainly thrived in the dysoxic mesopelagic zone below the surface mixed layer, while the marginal, high-latitude Neograptina inhabited oxic waters in the photic zone (*18*). During the LOME, the previously dominant Diplograptina experienced a severe decline in biodiversity, while the Neograptina not only survived the crisis but ultimately diversified to become the dominant species in early Silurian oceans (*16*–*21*). This selectivity implies a link between oceanic redox conditions and the reshaping of the pelagic marine ecosystem during the Ordovician-Silurian (O-S) transition.

The extinction of the mesopelagic Diplograptina has traditionally been attributed to improved ocean oxygenation resulting from increased ocean ventilation driven by the Hirnantian glaciation (*22*). However, recent studies, including from South China (*10*, *23*) and other global sections (*11*–*13*, *24*), suggest fluctuating and episodic expansion of ocean anoxia before the Hirnantian glaciation, challenging the extinction mechanism for the Diplograptina fauna. It has also been suggested that a decrease in nutrient supply from the deep ocean, because of changing ocean circulation patterns, may have been responsible for the observed biodiversity decline in graptolite communities during the LOME (*18*). However, marine nutrient cycling during the LOME remains poorly constrained, particularly regarding its potential influence on changes in the graptolite community structure.

Phosphorus (P) is generally considered the ultimate limiting nutrient for the growth of primary producers on geologic timescales (*25*). Given that primary producers were a food source for graptolites (*26*), P bioavailability may have been a crucial factor in the decline and recovery of graptolite diversity. Redox-related (bio)geochemical cycling of iron (Fe) plays a critical role in the regulation of dissolved P concentrations and, thus, P bioavailability in marine systems (*27*). Specifically, under oxic water column conditions, P is typically delivered to the sediment via the burial of organic matter and/or uptake in association with Fe (oxyhydr)oxides (Fe_ox_) (*28*, *29*). Once in the sediments, a portion of the P may be released to porewaters via the remineralization of organic P (P_org_) and the reductive dissolution of Fe_ox_ (*30*). However, the released P can readsorb to Fe_ox_ close to the sediment-water interface (*28*) or may precipitate as carbonate fluorapatite (*31*). Thus, P recycling back to the water column under oxic bottom water conditions is limited.

Under anoxic ferruginous (Fe^2+^-containing, with limited sulfide) bottom waters, there is potential for extensive drawdown of P through uptake to Fe minerals formed in the water column, and this P may be trapped in the sediments, leading to a negative productivity feedback (*32*). Under euxinic (sulfidic) water column conditions, however, P recycling back to the water column may be particularly intensive during settling and early diagenesis (thereby increasing bioavailable P in the oceans) (*33*). This recycling is caused by the release of P during the reductive dissolution of Fe_ox_ (*34*), coupled with preferential release of P during organic matter degradation, which results in the generation of high molar organic C/P ratios relative to the Redfield ratio (106/1) (*23*, *30*). P recycling may also be enhanced because of limited formation of authigenic P minerals (e.g., carbonate fluorapatite and vivianite) when dissolved sulfide is present (*35*, *36*). However, the detailed impact of ocean redox and associated changes in Fe cycling and P bioavailability on the evolution of graptolites during the O-S transition is currently unstudied.

To address this issue, we apply Fe speciation, redox-sensitive trace element (RSTE) systematics, and Fe isotopes to shelf sediments from Gondwana (Shuanghe, South China) and deep-ocean sediments from Iapetus (Dob’s Linn, Scotland) across the O-S transition. Fe speciation is a widely applied proxy for distinguishing between oxic, ferruginous, and euxinic conditions (*37*, *38*). Complementary to this, the enrichment patterns of RSTEs (e.g., U, Mo, and V) in sediments provide an independent constraint on overlying water column conditions, owing to their distinct solubilities under different redox conditions (*39*). Furthermore, the Fe isotope composition of marine sediments is generally controlled by the influx of terrestrial Fe-bearing silicate minerals and the accumulation of highly reactive Fe phases [Fe_HR_; comprising carbonate-associated Fe (Fe_carb_), Fe_ox_, magnetite (Fe_mag_), and pyrite (Fe_py_)], which are regulated by redox-driven precipitation and remobilization between the water column and sediments (*40*, *41*).

If the redox structure in each ocean basin were similar, this integrated geochemical approach allows us to track the evolution of marine redox conditions and Fe cycling from a shelfal setting to a deep-ocean setting, providing a qualitative framework for evaluating P recycling during the O-S transition. To provide a quantitative constraint on P dynamics, we further use the phase partitioning of P using a sequential extraction scheme (*42*). These results are then interpreted in terms of controls on P bioavailability, thereby resolving the drivers of the selective extinction and the subsequent proliferation of graptolite communities across the O-S transition.

### Geological background

The northern margin of the South China block was flooded by a tropical epicontinental sea (Yangtze Shelf Sea) during the O-S transition and was in direct connection with the Panthalassa Ocean (fig. S1). We studied the Upper Ordovician–to–lower Silurian succession at Shuanghe (Yibin, South China), which is dominated by black shales formed in a deep inner-shelf setting (fig. S2) (*10*). The succession at Shuanghe can be correlated to the Wangjiawan section (Yichang, South China), the Global Boundary Stratotype Section and Point for the base of the Hirnantian Stage (*43*). The Dob’s Linn section (Southern Uplands, Scotland) is the Global Boundary Stratotype Section and Point for the O-S boundary (*44*) and consists of gray mudstones and black shales (fig. S3) that slowly accumulated on the abyssal plain of the Iapetus Ocean before incorporation into an accretionary prism on the eastern margin of Laurentia (*44*). Environmental and biotic events across the O-S transition are recorded in both sections, and their timing is well constrained by high-resolution graptolite zones (see text S1 for further details).

### Fe systematics

Marine sediments contain total iron (Fe_T_) in the form of silicate minerals [silicate-bound Fe (Fe_sil_)] and as phases that are considered highly reactive toward dissolved sulfide in near-surface environments (*37*, *38*, *45*). The Fe_HR_ pool is operationally defined via chemical extractions (see Materials and Methods) and comprises Fe_carb_, Fe_ox_, magnetite, and Fe_py_ (*38*, *46*). Sediments deposited beneath oxic bottom waters typically exhibit low Fe_HR_/Fe_T_ ratios (<0.22), while Fe_HR_/Fe_T_ ratios above 0.38 suggest an anoxic water column, and ratios between 0.22 and 0.38 are considered equivocal (*37*, *38*). In sediments deposited under anoxic conditions, the Fe_py_/Fe_HR_ ratio distinguishes between ferruginous (Fe_py_/Fe_HR_ < 0.6) and euxinic (Fe_py_/Fe_HR_ > 0.6 to 0.8) conditions (*38*). More specifically, Fe_py_/Fe_HR_ ratios between 0.6 and 0.8 suggest potentially euxinic conditions (*37*, *38*), and following suggested procedures (*38*), we provide further support for redox conditions by using RSTE enrichment factors (EFs; see Materials and Methods).

The Fe_sil_ fraction of marine sediments is considered to have a relatively constant δ^56^Fe value (0.09 ± 0.05‰ relative to the IRMM-014; see Materials and Methods for analytical details) (*40*) near that of terrestrial igneous rocks. By contrast, Fe_HR_ phases commonly display variable δ^56^Fe values because of notable isotopic fractionation during different mineral precipitation pathways (*41*). In particular, sedimentary Fe_py_ may have δ^56^Fe values ranging from −4 to +4‰ (*47*–*49*), with equilibrium isotopic fractionation between Fe(II)_(aq)_ and Fe_py_ [Δ^56^Fe_Fe(II)aq–py_] varying from −0.5 to +0.5‰ (see the Supplementary Materials for further details).

## RESULTS

### Redox reconstruction

Redox conditions were initially reconstructed via a combination of Fe speciation and RSTE (U, Mo, and V) systematics, and three distinct intervals are identified in both the Shuanghe and Dob’s Linn sections (see text S2 for further details). These intervals are broadly coeval and can be correlated between the two sections on the basis of graptolite zones.

**Fig. 1.**
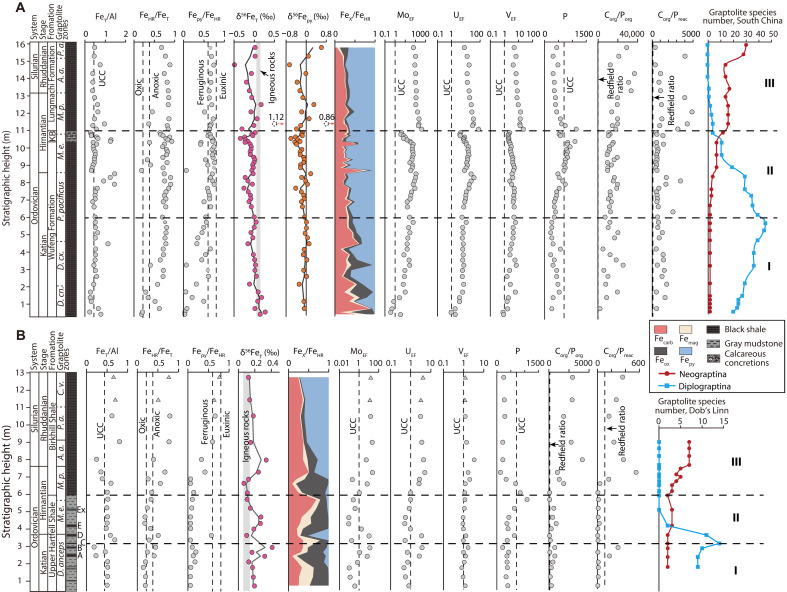
Chemostratigraphic profiles from shelf to deep ocean during the Ordovician-Silurian transition. (**A**) Shuanghe shelf section. (**B**) Dob’s Linn deep-ocean section. Fe speciation, P phase partitioning, and RSTE data for the Shuanghe section are from ref. (*10*). Diversity curves of the Diplograptina [Dicranograptidae-Diplograptidae-Orthograptidae or DDO fauna (*76*)] and Neograptina (Normalograptidae and its descendents or N fauna) in South China and Dob’s Linn are from refs. (*20*, *74*), respectively. Horizontal dashed lines separate the three geochemical intervals. Vertical dashed lines on Fe_T_/Al profiles represent average UCC (Fe_T_/Al = 0.44) (*50*). Fe_T_/Al and Fe speciation data for two samples (shown as triangles) are from ref. (*24*). Dashed lines on Fe_HR_/Fe_T_ profiles represent calibrated thresholds for distinguishing oxic (<0.22) and anoxic (>0.38) water column conditions. Dashed lines on Fe_py_/Fe_HR_ profiles represent the lower (0.6) and upper (0.8) thresholds for identifying euxinic conditions. Gray bands on δ^56^Fe_T_ profiles represent the igneous average value of 0.09 ± 0.05‰ (*40*). Black curves on δ^56^Fe_T_ and δ^56^Fe_py_ profiles represent the 10% LOWESS (locally weighted scatterplot smoothing) fit. Dashed lines on the P content plots represent the average UCC value of 700 ppm (parts per million). Dashed lines on molar C_org_/P_org_ and C_org_/P_reac_ plots represent the Redfield ratio of 106:1. Fe_T_/Al ratios and P contents are reported as wt %/wt % and ppm, respectively. Graptolite zones for the Shuanghe section are from refs. (*10*, *43*), and those for the Dob’s Linn section are from refs. (*44*, *86*). Graptolite-bearing shale bands A to Ex. on the Dob’s Linn lithostratigraphic column are from refs. (*44*, *86*). *D. cn.*, *Dicellograptus complanatus*; *D. cx.*, *Dicellograptus complexus*; *P. pacificus*, *Paraorthograptus pacificus*; *M. e.*, *Metabolograptus extraordinarius*; *M. p.*, *Metabolograptus persculptus*; *A. a.*, *Akidograptus ascensus*; *P. a.*, *Parakidograptus acuminatus*; *C. v.*, *Cystograptus vesiculosus*; *D. anceps*, *Dicellograptus anceps*; *Ex.*, *Extraordinarius*; KB, Kuanyinchiao Bed.

In interval I of the late Katian at Shuanghe, there is an overall increase in Fe_HR_/Fe_T_ ratios to values above the anoxic threshold, while Fe_py_/Fe_HR_ ratios mostly remain below the euxinic threshold (Fig. 1A). The RSTE EFs (see Materials and Methods for EF calculations) are consistent with Fe_HR_/Fe_T_ ratios, with increasing values through interval I, culminating in values that are considerably above the average upper continental crust (UCC) threshold (*50*). These observations suggest the progressive development of ferruginous conditions on the shelf setting (*10*). However, at Dob’s Linn, the base of interval I is characterized by predominantly oxic bottom waters, as indicated by low Fe_HR_/Fe_T_ ratios and very low Mo_EF_ and U_EF_ values (i.e., <1; Fig. 1B). In the upper portion of interval I, a shift toward intermittently ferruginous conditions is suggested by a fluctuating rise in Fe_HR_/Fe_T_ ratios to above the anoxic threshold, along with elevated RSTE EFs [particularly Mo_EF_ and U_EF_, both well above 1; see also ref. (*24*)].

In interval II of the late Katian-early Hirnantian, most Fe_HR_/Fe_T_ values from Shuanghe fall above the anoxic threshold (Fig. 1A). Fe_py_/Fe_HR_ values are generally high, with occasional values falling below the lower euxinic threshold of 0.6. Such values suggest that water column conditions were predominantly euxinic, with occasional transitions to ferruginous (or possibly dysoxic) conditions (*24*, *38*). Elevated RSTE EFs are also entirely consistent with deposition beneath anoxic bottom waters, with particularly high Mo_EF_ values (up to 123) supporting water column euxinia (see text S2 for further details). Notably, however, at the top of interval II, Mo_EF_ and U_EF_ values show a marked decline to below 10, coincident with subtle decreases in Fe_HR_/Fe_T_ and Fe_py_/Fe_HR_ ratios. This shift likely indicates a weakening of ocean euxinia and a transition to ferruginous conditions.

The decrease in the intensity of reducing conditions in the shelf setting toward the top of interval II is likely linked to enhanced ocean circulation, driven by the Hirnantian glaciation, which may have introduced O_2_-rich, polar-derived cold waters onto previously anoxic shelves (*10*, *13*, *51*). By contrast, at the oceanic Dob’s Linn section, most Fe_HR_/Fe_T_ values fall below the anoxic threshold, with low Fe_py_/Fe_HR_ values (with the exception of one sample that has a high value toward the bottom of this interval) and generally low RSTE EFs (Fig. 1B). In addition, a Mo_EF_ versus U_EF_ cross-plot shows that most samples from interval II fall in the oxic field (fig. S4). These results suggest that the Iapetus Ocean was dominated by oxic (or potentially dysoxic) conditions during interval II, with the exception of possible transient ferruginous conditions toward the base (*24*).

In interval III (late Hirnantian to early Rhuddanian), persistently high Fe_HR_/Fe_T_ and Fe_py_/Fe_HR_ ratios develop in the Shuanghe section, along with substantially elevated Mo_EF_ values (>69; mean, 170; table S1), suggesting persistent shelfal euxinia (Fig. 1A). At Dob’s Linn, however, while the base of this interval is marked by an overall increase in Fe_HR_/Fe_T_ values and moderately elevated RSTE EFs (generally <5), Fe_py_/Fe_HR_ values remain below the euxinic threshold (Fig. 1B), indicating the initial development of ferruginous bottom-water conditions. Persistently higher Fe_py_/Fe_HR_ ratios combined with highly elevated RSTE EFs in the Rhuddanian then suggest the more protracted development of euxinia in deeper waters (*12*, *24*). This interpretation is further supported by Mo_EF_-U_EF_ systematics (fig. S4), whereby samples from interval III generally exhibit significantly higher Mo_EF_ values relative to U_EF_ values, plotting within the euxinic field (*52*). This aligns with observations of pronounced Mo enrichment in sediments deposited under euxinic water column conditions (*53*, *54*). Notably, compared to the coeval Shuanghe section, Mo_EF_ values for the euxinic Dob’s Linn samples in interval III are lower (<30; mean, 15), likely reflecting weaker euxinia in the deep ocean (*24*, *55*).

### Fe isotope systematics

The evolution of marine redox conditions is further elucidated by bulk Fe isotope (δ^56^Fe_T_) and Fe_py_ isotope (δ^56^Fe_py_) records. In interval I at Shuanghe, δ^56^Fe_T_ transitions to slightly negative values after initially being close to the igneous average (*40*), consistent with progressive ocean anoxia on the Yangtze Shelf (Fig. 1A). At Dob’s Linn, δ^56^Fe_T_ and Fe_T_/Al profiles also show significant shifts that coincide with changes in redox conditions. In particular, as intermittent ferruginous conditions developed in the upper part of interval I, δ^56^Fe_T_ values greatly exceed the igneous range, coincident with decreasing Fe_T_/Al ratios to values that are considerably depleted relative to the UCC average. Notably, δ^56^Fe_T_ values shift to negative values at Shuanghe in response to more reducing conditions, whereas they rise to positive values at Dob’s Linn. These contrasting Fe-isotope signatures reflect distinct Fe behavior between shelf and deep-ocean settings, as discussed below.

In interval I, although Fe_py_ is generally the dominant Fe_HR_ phase at Shuanghe (Fig. 1A), δ^56^Fe_T_ shows only a weak negative correlation with Fe_py_/Fe_T_ (*R*^2^ = 0.24) and no clear correlation with δ^56^Fe_py_ (fig. S5). The wide range of Fe_py_ framboid sizes (fig. S6) suggests that most Fe_py_ was formed in sulfidic porewaters (see text S2 for further details) (*56*, *57*). The Fe^2+^ required for Fe_py_ formation likely originated from Fe^2+^ diffusion into the sediments as the overlying seawater transitioned to a ferruginous state, with a possible additional contribution from reductive dissolution of reactive Fe_ox_ in the sediments (*58*, *59*). Although Fe isotope fractionations during Fe_py_ precipitation can vary widely (−0.5 to +0.5‰; text S2), δ^56^Fe_py_ values in this interval vary only narrowly (−0.28 to 0.12‰). Moreover, the lowest δ^56^Fe_py_ value is above the typical δ^56^Fe range (−3.5 to −0.5‰) for Fe^2+^ generated by the reductive dissolution of Fe_ox_ (*59*), suggesting that this pathway made a limited contribution to diagenetic Fe_py_ formation. We thus propose that the diffusion of Fe^2+^ from the overlying ferruginous water column supplied most of the Fe for early diagenetic Fe_py_ formation, resulting in relatively stable δ^56^Fe_py_ values that contributed little to the observed variability in δ^56^Fe_T_.

The Fe_carb_ pool is the second most abundant Fe_HR_ fraction in this interval (Fig. 1A), and there is a distinct negative correlation between Fe_carb_/Fe_T_ and δ^56^Fe_T_ (*R*^2^ = 0.51; fig. S5). We suggest that part of the Fe_carb_ was precipitated from the ferruginous water column (*60*), with preferential incorporation of isotopically light Fe (*61*, *62*), leading to slightly negative δ^56^Fe_T_ values observed in interval I at Shuanghe. By contrast, at Dob’s Linn, Fe_ox_ are the dominant Fe_HR_ pool in interval I, and strongly positive δ^56^Fe_T_ values toward the top of this interval coincide with particularly low Fe_T_/Al ratios (Fig. 1B). This suggests that the development of intermittent deep-ocean anoxia resulted in reductive dissolution of reactive Fe_ox_ in sediments. However, low dissolved sulfide and bicarbonate concentrations in the deep-ocean sediments (indicated by low Fe_carb_/Fe_HR_ and Fe_py_/Fe_HR_ ratios; Fig. 1B) (*24*) appear to have allowed the preferentially released isotopically light Fe^2+^ (*61*–*63*) to diffuse readily into the overlying water column. This interpretation is supported by an overall negative correlation between Fe_T_/Al and δ^56^Fe_T_ (*R*^2^ = 0.52; fig. S7). Consequently, δ^56^Fe_T_ values increase to strongly positive values in response to the loss of isotopically light Fe from sediments under more reducing conditions.

Interval II shows two distinct negative δ^56^Fe_T_ shifts at Shuanghe (Fig. 1A). The first shift, in the upper *Paraorthograptus pacificus* zone, shows a progressive trend toward lighter δ^56^Fe_T_ compositions as Mo_EF_ values increase, which likely reflects a gradual shift from weakly to highly euxinic conditions. Subsequently, the second shift, in the upper *Metabolograptus extraordinarius* zone, again documents a progressive decrease in δ^56^Fe_T_ as Mo_EF_ values increase, suggesting consistent behavior with regard to the response of δ^56^Fe_T_ to the intensity of euxinia. This is confirmed at the top of interval II, where δ^56^Fe_T_ compositions return to higher values as Mo_EF_ drops to values that are only slightly elevated, suggestive of a return to weakly euxinic conditions at most. The similarity between the δ^56^Fe_T_ and δ^56^Fe_py_ profiles (Fig. 1A), coupled with an overall positive correlation between δ^56^Fe_T_ and δ^56^Fe_py_ (*R*^2^ = 0.53; fig. S8), in interval II suggests that the former is dominantly controlled by the relative extent of water column Fe_py_ formation under varying intensities of euxinia.

Interval II in the Dob’s Linn section has overall heavier δ^56^Fe_T_ values relative to the Shuanghe section, with the second positive δ^56^Fe_T_ excursion occurring in the middle (Fig. 1B). The generally heavier δ^56^Fe_T_ values likely reflect noneuxinic conditions in the deeper ocean at this time, whereby in contrast to the Shuanghe section, the δ^56^Fe_T_ profile was not controlled by the precipitation of isotopically light δ^56^Fe_py_ (Fe_py_ contents are generally very low in this interval). The positive δ^56^Fe_T_ excursion in the middle of interval II occurs coincident with the expansion of deep-ocean oxygenation during the Hirnantian glaciation (*10*, *13*, *51*) and is distinct from the positive excursions that occur elsewhere in the Dob’s Linn section, in that it is not associated with anoxic deeper water conditions or low Fe_T_/Al ratios (Fig. 1B). This particular excursion may be explained by precipitation of Fe_ox_ (preferentially incorporating isotopically heavy Fe) (*64*, *65*) during the widespread transition to better oxygenated conditions.

The establishment of widespread euxinia after the Hirnantian glaciation is recorded by Fe isotopes in interval III. At Shuanghe, δ^56^Fe_T_ initially increases to values close to the average igneous range before decreasing and then increasing again up-section (Fig. 1A). As with interval II, this trend is closely mirrored by the δ^56^Fe_py_ data. However, in contrast to interval II, the trends in δ^56^Fe_T_ and δ^56^Fe_py_ do not appear to relate to the intensity of euxinia, and instead, the persistent development of highly euxinic conditions is indicated by very high enrichments in Mo (Fig. 1A; see Discussion below). At Dob’s Linn, δ^56^Fe_T_ values remain within or above the igneous range throughout interval III, with a positive δ^56^Fe_T_ excursion that, as with interval I, coincides with decreased Fe_T_/Al ratios, suggesting the loss of Fe^2+^ to the overlying water column under ferruginous conditions. Following the subsequent transition from ferruginous to weakly euxinic conditions (*12*, *24*), Fe_T_/Al ratios increase above the UCC average, consistent with enhanced drawdown of Fe^2+^ in the form of Fe_py_, which in turn resulted in lighter δ^56^Fe_T_ values. However, the development of only weak euxinia in the deep ocean (as indicated by low Mo_EF_ values relative to the Shuanghe section; Fig. 1) resulted in δ^56^Fe_T_ values that remained isotopically heavier than at Shuanghe.

## DISCUSSION

### Deep-ocean sedimentary Fe release

Our data reveal ferruginous anoxia in the deeper and shallower oceans during the Late Ordovician to Early Silurian. Two primary sources may have potentially contributed dissolved Fe^2+^ to the global ocean: hydrothermal vent inputs and benthic Fe release from anoxic marine sediments (*66*, *67*). Hydrothermal fluids are considered a major Fe^2+^ source during certain intervals of the Precambrian, but their contribution was likely more limited in the more oxygenated Phanerozoic ocean (*66*). Sediment-derived Fe^2+^ primarily arises from the reductive dissolution of reactive Fe_ox_ and is characterized by low δ^56^Fe values (−3.5 to −0.5‰) (*59*, *68*). Providing this dissolved Fe^2+^ does not react with additional Fe_ox_, dissolved sulfide, or bicarbonate in pore waters, it may diffuse into the overlying water column (*41*). In the modern ocean, such Fe^2+^ release mainly occurs in O_2_-depleted continental shelf settings, where it is subsequently transported within the O_2_ minimum zone to the ocean interior (*69*, *70*). Such shelf-to-basin Fe shuttling has also been documented during Cretaceous oceanic anoxic events, contributing significantly to oceanic Fe cycling (*71*, *72*).

**Fig. 2.**
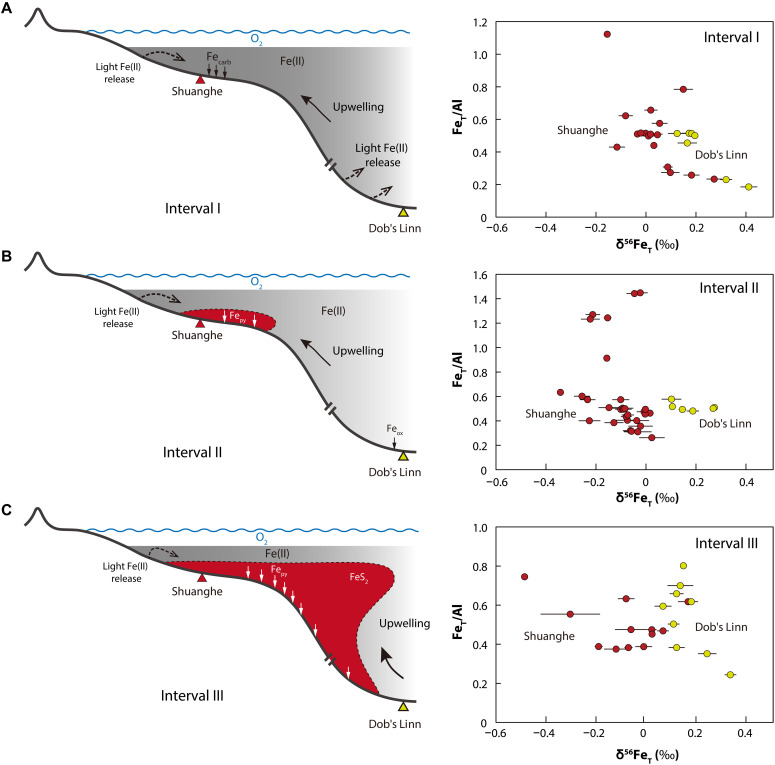
Oceanic Fe cycling during the O-S transition, with cross plots of Fe_T_/Al versus δ^56^Fe_T_ for the three geochemical intervals. (**A**) Interval I: Precipitation of Fe_carb_ under ferruginous shelf conditions, accompanied by the release of isotopically light Fe from anoxic sediments on the proximal shelf and in the deep ocean. (**B**) Interval II: Burial of Fe_py_ during episodes of shelf euxinia, concurrent with the precipitation of Fe_ox_ in dysoxic to oxic deep-ocean environments. (**C**) Interval III: Expansion of euxinic waters following the termination of the Hirnantian glaciation, leading to widespread Fe_py_ burial across both shelf and deep-ocean settings. Red and yellow filled circles denote samples from the Shuanghe and Dob’s Linn sections, respectively. The error bars indicate the 2σ uncertainty of δ^56^Fe_T_ values.

**Fig. 3.**
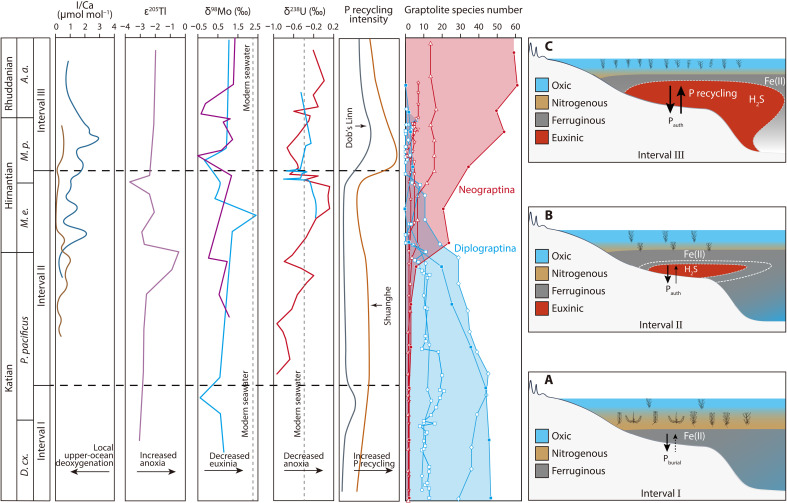
Generalized chemostratigraphic trends, relative intensity of P recycling, biodiversity changes, and conceptual model for graptolite evolution across the O-S transition. (**A**) During the late Katian (interval I), the expansion of anoxic seawater promoted the proliferation of the Diplograptina graptolites. (**B**) The intensification of oceanic anoxia, with episodic intrusion of toxic hydrogen sulfide (interval II), resulted in the contraction and loss of nitrogenous habitats for the previously dominant Diplograptina during the latest Katian-early Hirnantian. (**C**) The previously marginal Neograptina radiated in nutrient-rich oxygenated waters as a result of enhanced P recycling during the late Hirnantian-early Rhuddanian (interval III). The I/Ca profile is from ref. (*87*). The ε^205^Tl profile is from ref. (*11*). The δ^98^Mo profiles are from ref. (*12*) (purple line) and ref. (*88*) (blue line). The δ^238^U profiles are from ref. (*89*) (blue line) and ref. (*90*) (red line). Global graptolite biodiversity changes (red-filled squares and blue-filled circles) are from ref. (*76*), data for South China (open diamonds and triangles) from ref. (*20*), the Vinini Creek section (open squares and inverted triangles) from ref. (*16*), and the Dob’s Linn section (open hexagons and stars) from ref. (*74*). Full names of graptolite zones are provided in the caption for Fig. 1.

Shelf-to-basin Fe shuttling typically results in elevated Fe_T_/Al ratios and decreased δ^56^Fe_T_ values in deeper ocean sediments (*69*, *70*). However, our δ^56^Fe_T_ values from the deep-ocean Dob’s Linn section are consistently higher than those from the Shuanghe section across all intervals (Fig. 2). Notably, as discussed above, the low Fe_T_/Al ratios coincident with high δ^56^Fe_T_ values at Dob’s Linn suggest the loss of isotopically light Fe from the deposited sediments under ferruginous conditions. However, deep-ocean sediments were unlikely to constitute a continuous Fe^2+^ source throughout the three intervals. Consistently stable Fe_T_/Al values above the UCC average in the lower-middle part of interval I and the middle-upper part of interval II imply that most upward-diffusing Fe^2+^ was reoxidized and deposited under oxic conditions close to the sediment-water interface, resulting in minimal Fe loss from these samples. Similarly, following the expansion of global euxinia after the Hirnantian glaciation (as indicated by recent studies using ε^205^Tl, δ^98^Mo, and δ^238^U data; Fig. 3), the elevated Fe_T_/Al ratios and decreased δ^56^Fe values in the upper part of interval III at Dob’s Linn reflect the enrichment of Fe_py_ in the sediments (i.e., the deep-ocean sediments transitioned from being a source to a sink of Fe^2+^). These results indicate that Fe^2+^ release from deep-ocean sediments was primarily associated with ferruginous bottom-water conditions, whereas euxinic and oxic bottom waters predominantly facilitated the trapping and burial of Fe^2+^.

Thus, while an additional flux of Fe^2+^ from hydrothermal venting cannot be discounted, and Fe^2+^ may also have been sourced via the shallow shelf-to-basin shuttle (*69*–*72*), we propose that deep-ocean sediments served as a significant source of dissolved Fe^2+^ in ferruginous Early Paleozoic oceans, whereas mid-shelf areas primarily acted as an Fe^2+^ sink. This mechanism is particularly pronounced in interval III, where the increased isotopically light Fe^2+^ sourced from deep-ocean sediments during the development of ferruginous conditions was transported to continental shelf margins via vigorous upwelling during the early Silurian (*23*, *51*). This resulted in decreased seawater δ^56^Fe compositions in shelf settings, which contributed to the particularly low δ^56^Fe_py_ values observed around the *Akidograptus ascensus* graptolite zone at Shuanghe (Fig. 1A).

### Redox-driven P recycling

In the Shuanghe section, P contents remain below the UCC average throughout interval I (Fig. 1A), whereas molar ratios of both organic carbon (C_org_) to P_org_ and C_org_ to reactive P (P_reac_; representing potentially bioavailable P phases; see Materials and Methods) are considerably higher than the Redfield ratio (106:1). This suggests effective P recycling to the water column (*23*, *55*) in association with the progressive development of anoxia. Notably, although the sediments were deposited under ferruginous conditions, sulfide production during diagenesis (as reflected by relatively high Fe_py_/Fe_HR_ ratios; Fig. 1A) promoted P recycling (*23*, *73*). In the Dob’s Linn section (Fig. 1B), C_org_/P_org_ ratios above the Redfield ratio in interval I suggest the preferential release of P during organic matter remineralization (*23*, *32*, *55*). However, persistent very low C_org_/P_reac_ ratios suggest that the released P was largely retained in the sediment as authigenic phases through sink switching (*28*, *31*, *32*). An exception occurs during deeper-ocean anoxia at the top of interval I, where the C_org_/P_reac_ ratio of two samples exceeds the Redfield ratio (Fig. 1B). This coincides with positive shifts in δ^56^Fe_T_ values, reflecting the loss of light Fe^2+^ resulting from reductive dissolution of Fe_ox_, which promoted the release of Fe-bound P and its subsequent recycling to the water column (*28*, *31*).

During interval II at Shuanghe, P recycling continued, as indicated by C_org_/P_reac_ ratios above the Redfield ratio (Fig. 1A). However, the extent of P recycling to the deep ocean was significantly diminished, as indicated by elevated P concentrations and consistently low C_org_/P_reac_ ratios at Dob’s Linn (Fig. 1B). This is consistent with dominantly oxic conditions in the deeper ocean during the Hirnantian glaciation (Fig. 3) (*10*, *13*), facilitating efficient drawdown and fixation of dissolved P via the precipitation of Fe_ox_ (*28*).

In interval III, coincident with the reestablishment of ocean euxinia (as indicated by significant excursions in ε^205^Tl, δ^98^Mo, and δ^238^U records; Fig. 3), more intensive P recycling occurred in both shelf and deep-ocean settings. This is evidenced at Shuanghe and Dob’s Linn by decreased P contents and highly elevated C_org_/P_reac_ ratios (Fig. 1). This enhanced P recycling is further supported by the positive δ^56^Fe_T_ and δ^56^Fe_py_ records at the base of interval III, which reflect efficient drawdown of dissolved Fe^2+^ in the form of Fe_py_ (*35*). In addition, P recycling back to the water column may be further intensified by rapid reduction of P-bearing Fe_ox_, preferential release of P from decaying organic matter during sulfate reduction, and inhibited formation of authigenic P minerals in euxinic sediments (*35*, *36*). Even under low-sulfate euxinia, a moderate degree of P recycling may occur, as observed in the modern Lake Cadagno, Switzerland (*73*). Such P cycling would have increased phosphate bioavailability in the ocean, promoting a positive productivity feedback and, hence, increased organic burial. This is supported by widespread deposition of organic-rich shales during the Early Silurian (*13*, *23*, *24*), suggesting the proliferation of primary producers in surface waters.

### Implications for graptolite evolution across the O-S transition

During the late Katian (interval I and the lower half of interval II), Diplograptina was the dominant graptolite group in the global ocean, as indicated by fossil records from Vinini Creek (NV, US) (*16*), South China (*20*), and Dob’s Linn sections (Figs. 1 and 3) (*74*). This is consistent with progressive ocean deoxygenation (Fig. 3A), which would have facilitated the proliferation of graptolite species living in O_2_-depleted, nitrogenous (denitrifying) waters (*18*, *22*, *75*) and their subsequent preservation in sediments (*26*). However, during the early-middle Hirnantian stage (upper half of interval II), graptolite fossils are nearly completely absent at Shuanghe, and the diversity of the previously dominant Diplograptina also declines markedly in South China (*20*), Vinini Creek (*16*), Dob’s Linn (*74*), and other contemporaneous sections (Fig. 3) (*76*). This diversity loss is simultaneous with the intensified development of reducing conditions, from ferruginous to euxinic, at Shuanghe (Fig. 3B). The apparent temporal correlation between these redox perturbations and the decline in graptolite diversity highlights the possibility that dynamic marine euxinia, rather than oxygenation, played a major role in driving the extinction of the Diplograptina during the Late Ordovician. It is plausible that the episodic expansion and diffusion of toxic sulfide from the seafloor could have accounted for a diminished nitrogenous zone, thereby resulting in the gradual demise of the Diplograptina graptolites inhabiting this zone.

By contrast, the previously marginal Neograptina, which preferentially inhabited fully oxygenated surface waters of the photic zone, was not significantly affected. Notably, the species diversity of the Neograptina increased concurrently with the gradual extinction of the previously dominant Diplograptina graptolites during the latest Katian and Hirnantian stages (Fig. 3) (*16*, *18*, *20*, *74*, *76*). In particular, both the species diversity and abundance of the Neograptina significantly increased during the late Hirnantian, coincident with the widespread and persistent development of ocean euxinia and enhanced P recycling (Fig. 3C). Such euxinia-promoted P recycling after the Hirnantian glaciation likely created highly eutrophic oceanic conditions (*23*, *33*, *77*), fueling the proliferation of primary producers in surface waters. The resultant increase in food supply likely supported the diversification of the Neograptina in the epipelagic zone, while the mesopelagic zone was uninhabitable (Fig. 3C). In addition, reduced ecological competition due to the extinction of the Diplograptina graptolites may have functioned as a secondary factor promoting the radiation of the Neograptina. We thus conclude that wide scale changes in Fe cycling, which were linked to transitions in global oceanic redox state and the consequent behavior of the P cycle, were ultimately responsible for both the initial demise and subsequent radiation of select graptolite species across the O-S transition.

## MATERIALS AND METHODS

A total of 84 samples were obtained from the Shuanghe section (South China) and Dob’s Linn section (Scotland) (see text S1 for further details). We analyzed Fe isotope compositions of 61 samples collected from the Wufeng-to-Lungmachi formations of the Shuanghe section (see table S1 for all data). Twenty-three samples were collected from the Upper Hartfell Shale and Birkhill Shale at Dob’s Linn for analyses of Fe speciation, Fe isotopes, trace elements (Mo, U, and V), and P phase partitioning (see tables S2 and S3 for all data).

### Elemental analyses

Elemental analyses of 21 samples from the Dob’s Linn section were conducted in the Cohen Geochemistry Laboratory, University of Leeds. Powdered samples were first ashed at 550°C for 8 hours, followed by total digestion using a combination of concentrated HNO_3_, HF, HClO_4_, and H_3_BO_3_ (*78*). Major element (Fe and Al) concentrations were measured using inductively coupled plasma optical emission spectrometry, and trace element (U, Mo, and V) concentrations were measured via inductively coupled plasma mass spectrometry (ICP-MS). Accuracy was ensured by replicate analyses of the international sediment standard SGR-1b, with a relative standard deviation (RSD) of <5% for all elements except Mo, which had an RSD of 12%. EFs for each element (X_EF_) were calculated relative to the average UCC (*50*) as

$$X_{\text{EF}}={\left(X/\text{Al}\right)}_{\text{sample}}/{\left(X/\text{Al}\right)}_{\text{UCC}}$$

### Fe speciation analyses

A total of 21 samples from the Dob’s Linn section were selected for Fe speciation analyses (*46*) in the Cohen Geochemistry Laboratory, University of Leeds. First, Fe carbonate phases (e.g., siderite and ankerite; Fe_carb_) were targeted with a sodium acetate solution at pH 4.5 and 50°C for 48 hours. Afterward, ferric (oxyhydr)oxides (e.g., ferrihydrite, goethite, and hematite; Fe_ox_) were targeted using a sodium dithionite solution at pH 4.8 and room temperature for 2 hours. Then, magnetite was extracted using an ammonium oxalate solution at room temperature for 6 hours. The concentration of Fe phases was subsequently determined by a Thermo Fisher Scientific iCE-330 Atomic Absorption Spectrometer. Accuracy was ensured by replicate analyses of the WHIT international sediment standard (*78*), with an RSD of <5% for all steps.

To allow the determination of Fe isotopes in Fe_py_, we used additional steps following on directly from the ammonium oxalate extraction (*79*), whereby dissolved Fe_sil_ was first extracted with a solution comprising 2 ml of concentrated HF and 10 ml of 50% HCl at room temperature for 1 hour. This dominantly leaves Fe_py_, which was extracted using concentrated HNO_3_ at room temperature for 2 hours. This combined sequential extraction technique for analyzing Fe isotopes in different Fe extracts has been successfully validated and used in studies of both modern marine sediments (*80*) and ancient rocks (*81*).

To quantitatively determine Fe_py_ via conventional methods, we used a two-step distillation procedure (*82*). Fe monosulfides (below detection in all cases) were initially extracted via a near-boiling 50% HCl extraction. Chromous chloride was then used to extract Fe_py_-bound sulfur. The liberated sulfide was trapped as Ag_2_S, and the concentration of extracted sulfide (and, hence, Fe_py_) was determined gravimetrically.

### Fe isotope analyses

A total of 61 samples from the Shuanghe section and 23 samples from the Dob’s Linn section were selected for bulk Fe isotope analyses. Fe_py_ isotope compositions were measured on extraction solutions (see above) for 53 samples from the Shuanghe section. Sample dissolutions (for bulk samples) and all chemical purification steps and Fe isotope analyses were performed at the State Key Laboratory of Geological Processes and Mineral Resources, China University of Geosciences, Wuhan.

Powdered samples were digested in a mixture of concentrated HNO_3_, HCl, and HF in a clean room. Fe was separated from its matrix via anion-exchange (AG MP-1) chromatography, whereby the matrix elements (e.g., Ca, Mg, and Na) were eluted in 12 ml of 7 M HCl, and Fe was collected in 10 ml of 1 M HNO_3_ after discarding the first 0.4 ml of eluent. Fe isotope ratios were measured by multicollector inductively coupled plasma mass spectrometry (MC-ICP-MS; Nu Plasma 1700) operated in high-resolution mode. The instrumental mass bias was corrected using a standard-sample bracketing approach. Samples and standards were prepared in a 0.35 M HNO_3_ solution and introduced to the Ar plasma via a Cetac ASX-112FR automatic sampler (CETAC Technologies, Omaha, NE, US) and a self-aspirating Glass Expansion Micromist nebulizer (Glass Expansion, Pocasset, MA, US), with an uptake rate of ~100 μl min^−1^. Compiled data for IRMM-014 and geological reference materials (e.g., BHVO-2, BCR-2, AGV-2, and DTS-1) over eight measurement sessions demonstrated that the external reproducibility for δ^56^Fe was better than 0.05‰ at the 2SD level. The Fe isotopic compositions of the samples are reported relative to the reference material IRMM-014 as

δiFe (‰)=[(Fie/F54e)sample/(Fie/F54e)IRMM−014−1]×1000

where i represents either ^56^Fe or ^57^Fe.

### P phase partitioning

P phase partitioning was determined for 23 samples from the Dob’s Linn section via an established sequential extraction method (*42*) in the Cohen Geochemistry Laboratory, University of Leeds. The method targets four operationally defined sedimentary P pools, including iron-bound P (P_Fe_), authigenic carbonate fluorapatite–associated P (P_auth_), crystalline apatite (P_cryst_), and organic-bound P (P_org_). The concentrations of P_auth_, P_cryst_, and P_org_ phases in wash solutions (MgCl_2_ and water) were measured by a spectrophotometer using the molybdate blue method (*83*), while the concentration of P_Fe_ was determined via inductively coupled plasma optical emission spectrometry. Replicate analyses of an international sediment reference material (WHIT) gave RSDs of <5% for each step. P_reac_ (P phases that are potentially bioavailable, expressed as P_reac_) was calculated as the sum of P_auth_, P_org_, and P_Fe_.

### RSTE systematics

RSTEs (e.g., U, Mo, and V) are valuable proxies for assessing redox conditions, owing to their distinct solubilities under varying redox states. Specifically, U and Mo typically occur in the dissolved forms of U(VI) and Mo(VI) under oxic water column conditions (*84*, *85*). Under anoxic conditions, U(VI) is reduced to insoluble U(IV), resulting in its enrichment in sediments (*84*). In the presence of H_2_S in the water column, Mo(VI) is transformed to particle-reactive thiomolybdate (MoO*_x_*S_4_^*x*−^), which can be significantly enriched in the sediments (*53*, *54*). Vanadium also behaves differently under varying redox conditions. Under oxic water column conditions, V exists predominantly as quasi-conservative V(V) and may be transported to sediments adsorbed onto Mn oxides (*54*). In dysoxic porewaters, where Mn oxides are reduced to Mn^2+^, V may be released, leading to its depletion in sediments (*39*, *54*). Under anoxic conditions, V(V) is reduced to surface-reactive V(IV), leading to its accumulation in sediments (*54*). However, the accumulation of RSTEs in sediments can also be influenced by other factors, such as terrestrial inputs and biogenic carbonate. Thus, to be able to compare RSTE enrichments in samples, EFs that normalize the concentration of a specific element X to the average UCC (*50*) are commonly calculated

$$X_{\text{EF}}={\left(X/\text{Al}\right)}_{\text{sample}}/{\left(X/\text{Al}\right)}_{\text{UCC}}$$

## Supplementary Materials

### This PDF file includes:

Supplementary Text S1 and S2 Figs. S1 to S8 Tables S1 to S3 References
