# Supplementary material for: Dynamic redox–promoted iron and nutrient cycling drove graptolite evolution across the Ordovician-Silurian transition
Source: Sci Adv. 2026 Jan 21;12(4):eaea1423. doi: 10.1126/sciadv.aea1423 (PMC12822635; doi:10.1126/sciadv.aea1423)
Supplement: Supplementary file 1 — Supplementary Text S1 and S2 Figs. S1 to S8 Tables S1 to S3 References [file sciadv.aea1423_sm.pdf]

Science Advances
AAAS

Supplementary Materials for

Dynamic redox–promoted iron and nutrient cycling drove graptolite evolution across the Ordovician-Silurian transition

Zhen Qiu *et al.*

Corresponding author: Zhen Qiu, qiuzhen316@163.com; Caineng Zou, zcn@petrochina.com.cn; Simon W. Poulton, s.poulton@leeds.ac.uk

Sci. Adv. **12**, eaea1423 (2026)
DOI: 10.1126/sciadv.aea1423

This PDF file includes:

Supplementary Text S1 and S2
Figs. S1 to S8
Tables S1 to S3
References

Supplementary Text

Text S1. Geological setting

South China was situated at a low latitude during the Ordovician-Silurian (O-S) transition (Fig. S1A), near the northern margin of Gondwana, and consisted of the Yangtze Block to the northwest and the Cathaysia Block to the southeast (*91*). The northern part of the Yangtze Block was flooded by the epicontinental Yangtze Shelf Sea, which deepened northwards and passed into the Panthalassa Ocean (Fig. S1B). Upper Ordovician to lower Silurian strata in the Yangtze region comprise the basal Pagoda and Linhsiang platform limestones of the early-mid Katian (*92*). The overlying late Katian strata comprise carbonaceous shales of the Wufeng Formation, which record regional sea-level rise and increased terrestrial inputs due to the northward progression of the Kwangsian Orogeny during the late Katian (*93*). The overlying Kuanyinchiao Bed (KB), consisting of calcareous mudstones with carbonate concretions and abundant shelly fossils of the cool-water *Hirnantia* fauna, accumulated during the Hirnantian glacial maximum (*94*). The succeeding graptolitic shale of the Lungmachi Formation deposited during post-glacial marine transgression, under euxinic oceanic conditions from the latest Hirnantian into the Rhuddanian (*10*).

The Shuanghe section (Yibin, Sichuan; 104.8842°E, 28.3847°N) was consistently situated in an inner-mid shelf setting during the late Katian (Fig. S1C). At Shuanghe, the basal 10 m of section comprises the Wufeng Formation, a black graptolitic shale and calcareous mudstone (Fig. S2). The overlying Kuanyinchiao Bed is about 60 cm thick and composed of calcareous mudstone and lenticular shelly mudstone. The overlying Lungmachi Formation consists of black, graptolitic shale > 10 m thick. In this study, we analyzed Fe isotope compositions of 61 samples collected from the Wufeng to Lungmachi formations (see Table S1 for all data), which span an interval of ~16 m. With the exception of two samples at the base of the Wufeng Formation (Fig. S2D; *59,95*), all other samples from the Shuanghe section are laminated or homogeneous (Fig. S2E), showing no evidence of bioturbation. This observation is consistent with our geochemical evidence, which indicates that the Wufeng and Lungmachi formations were dominated by anoxic bottom-water conditions that would have inhibited bioturbation. The elemental concentrations, Fe speciation and pyrite data for these samples have been reported in previous studies (*10,23,59*).

Dob's Linn is located in the Southern Uplands, Scotland (3°16′17.6″E, 55°25′46.5″N; Fig. S3A), and is the Global Stratotype Section and Point (GSSP) for the Ordovician and Silurian boundary (443.8 ± 1.5 Ma). The Late Ordovician to early Silurian succession at Dob's Linn belongs to the Moffatt Shale Group, which is subdivided into four formations in ascending stratigraphic order: the late Sandbian to early Katian Glenkiln Shale, the mid-Katian Lower Hartfell Shale, the late Katian to early Hirnantian Upper Hartfell Shale, and the latest Hirnantian to early Rhuddanian Birkhill Shale (*96*). The GSSP is defined at 1.6 m above the base of the Birkhill Shale, marked by the first appearance of *Parakidograptus acuminatus* (*52,97*).

In this study, we analyzed 23 samples collected from the uppermost 6 m of the Upper Hartfell Shale and the basal 7 m of the Birkhill Shale (see Table S2 for all data). The lower to middle portion (~23 m) of the Upper Hartfell Shale is mostly composed of non-graptolitic, pale grey/green mudstone, while the uppermost 5 m contains this lithology alternating with thin beds of black graptolitic shale, referred to as *Anceps* and *Extraordinarius* bands (*44*). Our petrographic analyses indicate that most samples are either homogeneous or laminated (Fig. S3E and S3F). A previous study (*98*) noted the localized presence of sub-millimetre scale meiofaunal burrows in the grey mudstones of the Upper Hartfell Shale (corresponding to intervals I and II in

our study), whilst the Birkhill Shale is laminated/unbioturbated. These observations tally with our geochemical evidence (see main test for details), which suggests that the unborrowed Interval III were deposited under weakly euxinic conditions, unfavorable for biological activity.

The *Anceps* bands are subdivided into five units, named A to E, with a total thickness of ~2 m (Fig. S3C). Anceps bands A and B are placed within the *Dicellograptus complexus* Zone, while bands C to D are assigned to the *Paraorthograptus pacificus* Zone. Anceps bands E and the *Extraordinarius* band belong to the Hirnantian *Metabolograptus extraordinarius* Zone. The basal Birkhill Shale is composed predominantly of black graptolitic shale with numerous thin bentonite seams. The early Rhuddanian *Akidograptus ascensus* Zone is defined at 1.6 m above the base of the Birkhill Shale (Fig. S3D), and spans an interval of 1.5 m. The top boundary of the *Parakidograptus acuminatus* Zone has not been precisely defined.

The Moffat Shale Group records condensed deep-marine deposition characteristic of an abyssal plain setting in the Iapetus Ocean (*98*), with an ultra-low sedimentation rate of <5 mm/ka (*24*). At Dob's Linn, the succession is structurally complex, having been tectonically disrupted by numerous minor and large-scale faults (Fig. S3B). As a result, this section is interpreted as part of an accretionary prism that developed along the southeastern margin of Laurentia during the progressive closure of the Iapetus Ocean in the Ordovician and Silurian (*99,100*).

Text S2. Pyrite Fe isotope geochemistry

Syngenetic pyrite formed in a euxinic water column is characterized by small-sized framboids (mean <5 μm) with a narrow size distribution (*59,101,102*). However, in the Shuanghe section, larger framboid sizes (mean 6.19 ± 2.2 μm; Fig. S6A) are observed in Interval I (*59*), suggesting that they mostly formed within the sediment. Therefore, we consider that there is likely a preferential expression of equilibrium isotopic fractionation and continuous isotopic exchange between this diagenetic pyrite and the $Fe(II)_{aq}$ in sedimentary pore fluids. Experimental studies (*103*) have demonstrated a kinetic isotope fractionation factor between $Fe(II)_{(aq)}$ and mackinawite (FeS, a pyrite precursor) of +0.85 ± 0.30‰, and an equilibrium fractionation factor of ~0.3‰. By contrast, equilibrium iron isotope fractionations ($\Delta^{56}Fe_{Fe(II)aq-FeS}$) of -0.52 ± 0.16‰ at 2°C and -0.33 ± 0.12‰ at 25°C have been reported (*104*). Similarly, Wu et al. (*105*) determined an equilibrium fractionation factor ($\Delta^{56}Fe_{Fe(II)aq-FeS}$) of -0.32 ± 0.29‰ at 20°C. By contrast, it has also been inferred that pyrite formation yields a larger kinetic isotope fractionation of up to +3‰ in the modern Black Sea water column (*106*). However, the most recent experiments (*48*) have revealed that the average isotopic fractionation during pyrite precipitation is -0.51 ± 0.22‰, while the rate-dependent kinetic isotope effect and/or isotopic exchange between pyrite and $Fe(II)_{aq}$ during pyrite precipitation can significantly influence $\delta^{56}Fe_{py}$ values (*107*). It is noteworthy, however, that these fractionation factors are independent of temperature, pH and precipitation pathways in low-temperature systems (*103,105*). Additionally, changes in the aqueous ferrous iron reservoir can also yield $\delta^{56}Fe$ variability in pyrite (*41*). Here, we estimate the equilibrium isotopic fractionation between $Fe(II)_{(aq)}$ and pyrite ($\Delta^{56}Fe_{Fe(II)aq-py}$) to be between -0.5‰ to +0.5‰, based on the combination of reported fractionation values (see above).

The massive burial of pyrite in Interval II is also supported by elevated sulfur isotope compositions of sedimentary pyrite ($\delta^{34}S_{py}$) in the Shuanghe section (Fig. S6B). A comparable increase in $\delta^{34}S_{py}$ values is also observed at Dob's Linn and other global sections (*13*). This observation is consistent with intensified bottom-water anoxia from ferruginous to euxinic conditions (*10,13*). In Interval III, the small size of pyrite framboids (mean <5 μm; Fig. S6A),

coupled with consistently decreased $\delta^{34}S_{py}$ values and high Fe_{py}/Fe_{HR} ratios, suggest that most pyrite was precipitated from a sulfide-rich water column. Given that the pyritization of dissolved aqueous ferrous iron in such conditions is near quantitative (*48,72*), it is likely that the $\delta^{56}Fe_{py}$ values of these Shuanghe samples closely approximate the $\delta^{56}Fe$ composition of contemporaneous seawater.

Fig. S1.

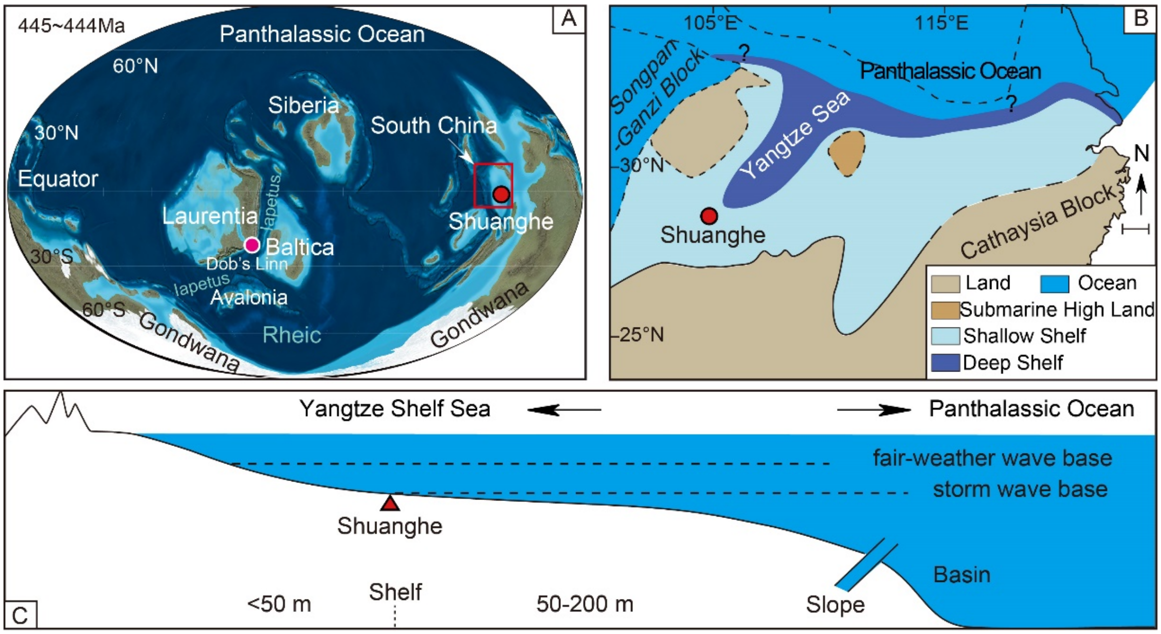

Fig. S1. Geological setting. (A) Late Ordovician (ca. 445~444 Ma) paleogeography, adapted from © 2020 Colorado Plateau Geosystems Inc.; **(B)** Simplified paleogeographic map of the Yangtze Shelf Sea showing the locality of the Shuanghe section during the Late Ordovician. Scale bar = 100 km; Figures from Zou et al. (2018; © The Geological Society of America, used with permission) **(C)** Schematic cross-section of the Late Ordovician Yangtze Shelf Sea (not to scale), showing estimated paleo-depth of the Shuanghe section (Figures from Zou et al. (2018; © The Geological Society of America, used with permission)).

Fig. S2.

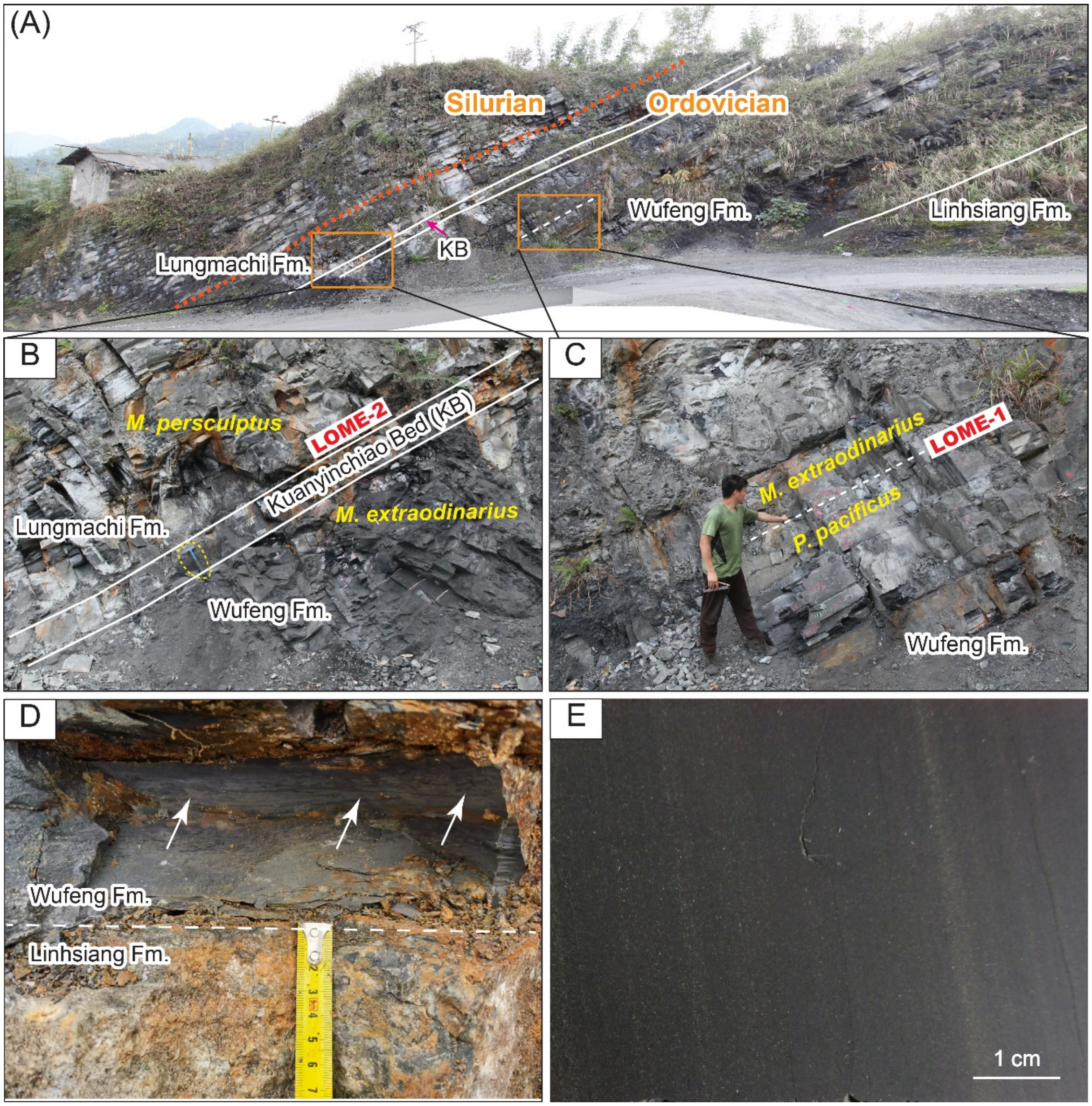

Fig. S2. (A) Panoramic view of the study section at Shuanghe, South China (Used with permission of Elsevier from ref. (59) permission conveyed through Copyright Clearance Center, Inc.); **(B-C)** Close-up of the strata boundaries in panel (A) (Used with permission of Elsevier from ref. (59) permission conveyed through Copyright Clearance Center, Inc.)). **(D)** Bioturbated gray mudstone at the base of the Wufeng Formation. **(E)** Banded-laminated shale in the Wufeng Formation (*59*). The graptolite zones are from refs. (*10,43*). *P. pacificus—Paraorthograptus pacificus*; *M. e.—Metabolograptus extraordinarius*; *M. p.—Metabolograptus persculptus*. LOME—Late Ordovician Mass Extinction.

Fig. S3.

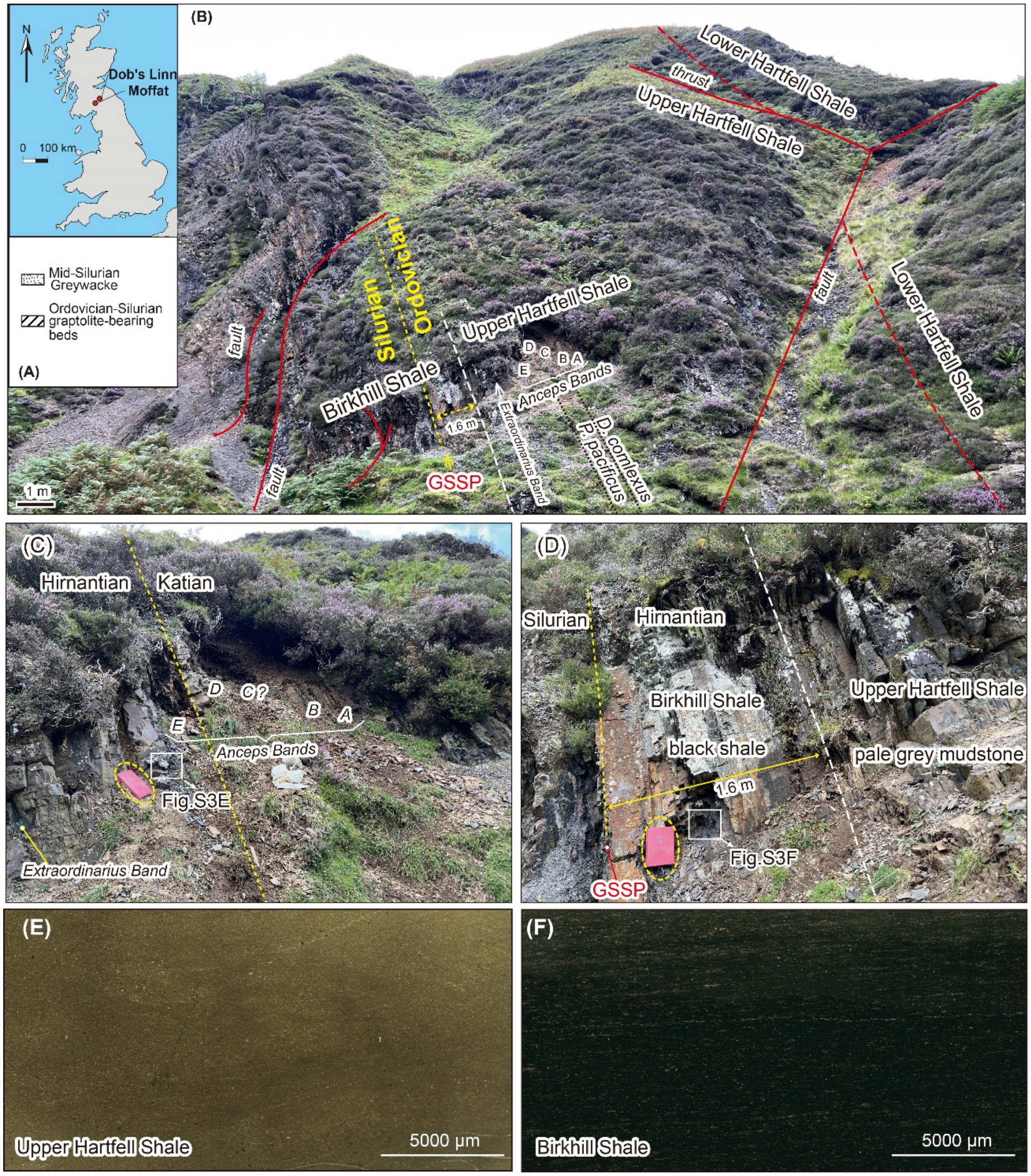

Fig. S3. **(A)** Location of the Ordovician–Silurian GSSP at Dob's Linn, Scotland, UK. **(B)** Panoramic photograph of the Linn Branch outcrop showing the transition from the Upper Hartfell Shale to the Birkhill Shale, graptolite zones, and the position of the GSSP. **(C)** Field photograph showing the *Anceps* and *Extraordinarius* Bands. **(D)** Field photograph showing the transition from the gray Upper Hartfell Shale to the black Birkhill Shale and the position of the O-S GSSP. The interpretation of graptolite zones is from ref. (*44,52*). Red notebook for scale (20 cm across). (E) Photomicrographs of homogeneous grey mudstone from the Upper Hartfell Shale (see panel C for the sampling location). (F) Photomicrographs of banded-laminated shales from the bottom of the Birkhill Shale (see panel D for the sampling position). *P. pacificus—Paraorthograptus pacificus*; *D. complexus.—Dicellograptus complexus*.

Fig. S4.

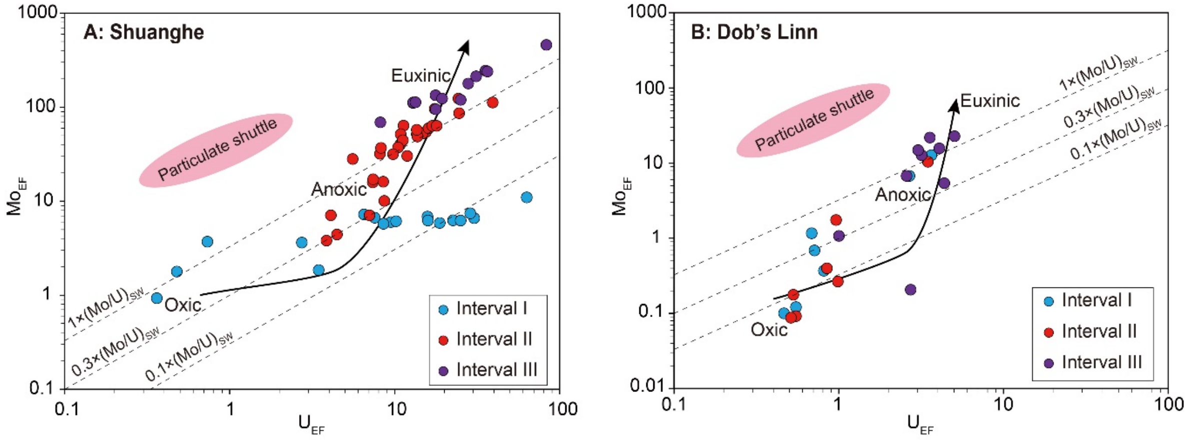

Fig. S4. Plots of Mo_{EF} vs. U_{EF} for samples from Shuanghe (A) and Dob's Linn (B). The dashed lines show the proportions of the seawater (SW) Mo/U molar ratio (*55*).

Fig. S5.

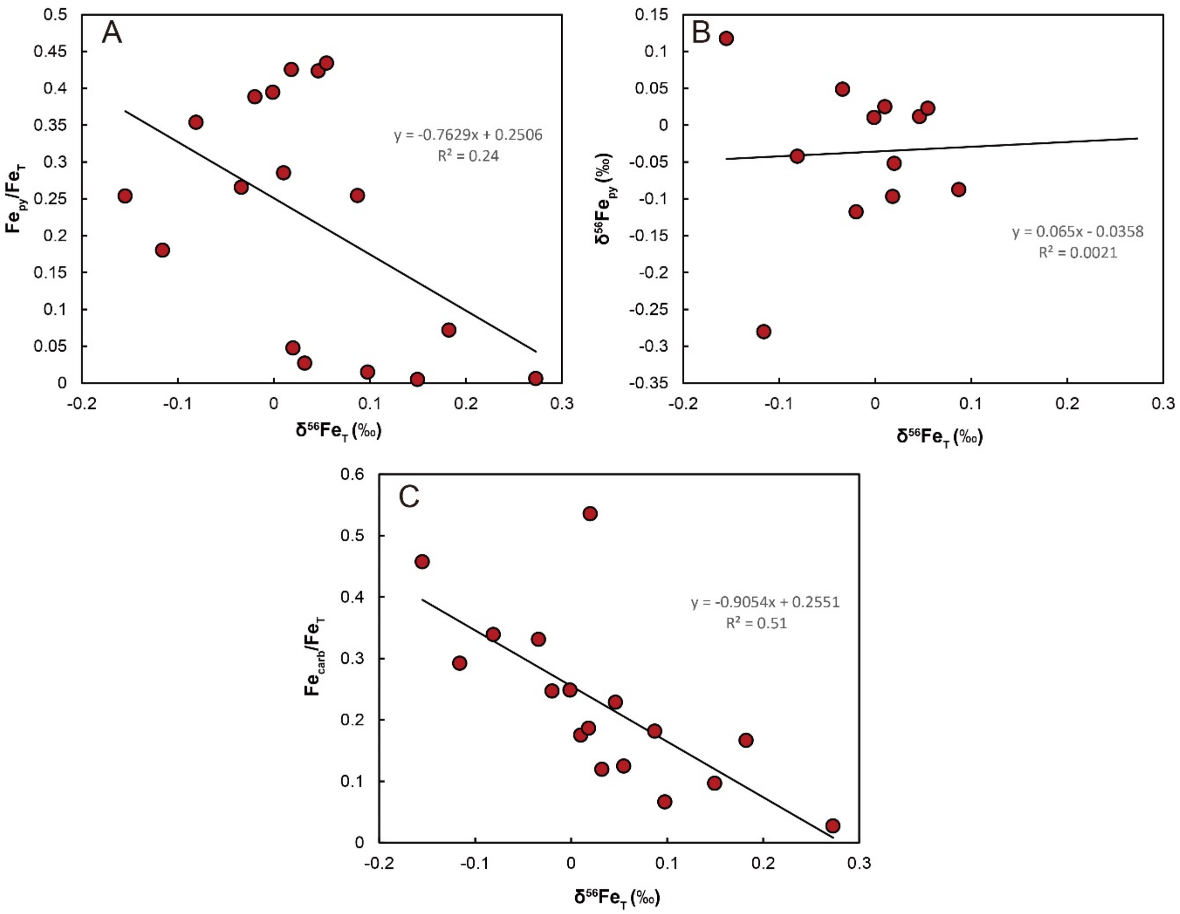

Fig. S5. Cross-plots of $\delta^{56}Fe_T$ vs Fe_{py}/Fe_T (A), $\delta^{56}Fe_T$ vs $\delta^{56}Fe_{py}$ (B), and $\delta^{56}Fe_T$ vs Fe_{carb}/Fe_T (C) for Interval I in the Shuanghe section.

Fig. S6.

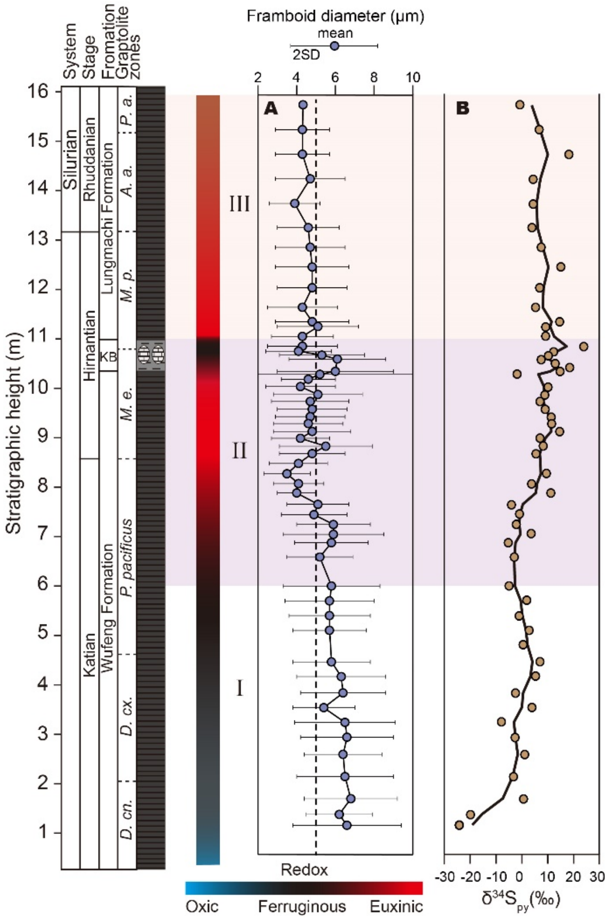

Fig. S6. Geochemical profiles for the Shuanghe section. (A) framboidal diameter. (B) pyrite sulfur isotopes ($\delta^{34}S_{py}$). Data are from ref. (*59*). Redox interpretations from ref. (*10*) and graptolite zones are from refs. (*10,43*). *D. cn.—Dicellograptus complanatus; D. cx.—Dicellograptus complexus; P. pacificus—Paraorthograptus pacificus; M. e.—Metabolograptus extraordinarius; M. p.—Metabolograptus persculptus; A. a.—Akidograptus ascensus; P. a.—Parakidograptus acuminatus.* KB—Kuanyinchiao Bed.

Fig. S7.

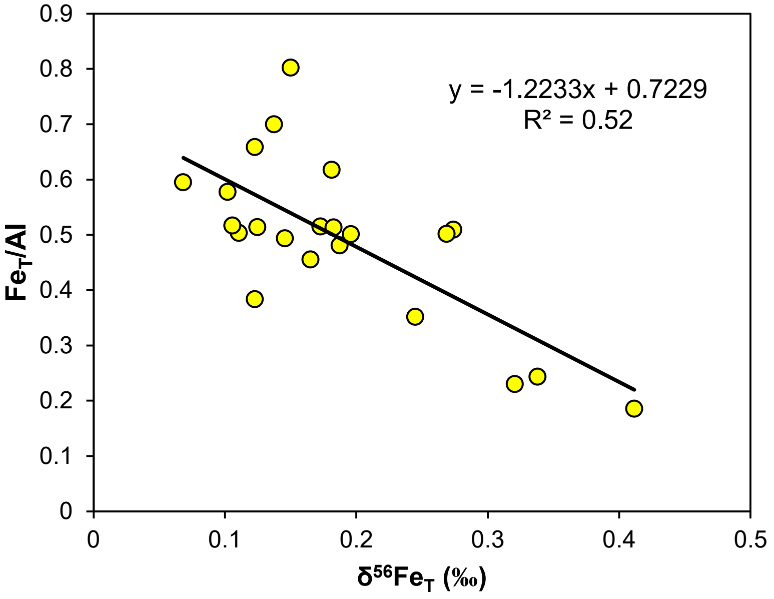

Fig. S7. Cross-plot of $\delta^{56}Fe_T$ vs Fe_T/Al for the Dob's Linn section.

Fig. S8.

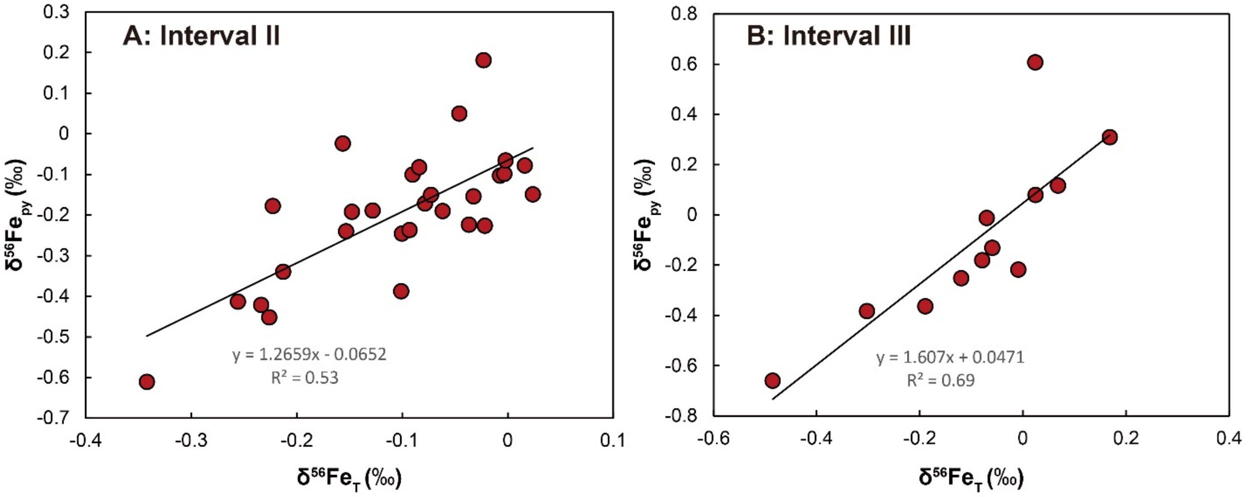

Fig. S8. Cross-plots of $\delta^{56}Fe_T$ vs $\delta^{56}Fe_{py}$ for Interval II (A) and Interval III (B) in the Shuanghe section.

Table S1. Total Fe isotope ($\delta^{56}Fe_T$) and pyrite Fe isotope ($\delta^{56}Fe_{py}$) data for the Shuanghe section, South China.

Height (m)	Interval	$\delta^{56}Fe_T$ (‰)	2SD	$\delta^{56}Fe_{Py}$ (‰)	2SD (‰)
15.79	III	0.02	0.02	0.61	0.01
15.29	III	0.02	0.01	0.08	0.03
14.79	III	-0.49	0.01	-0.66	0.03
14.29	III	-0.07	0.02	-0.01	0.05
13.79	III	-0.19	0.02	-0.36	0.02
13.3	III	-0.12	0.05	-0.25	0.05
12.9	III	-0.06	0.06	-0.13	0.04
12.5	III	0.17	0.04	0.31	0.03
12.08	III	-0.08	0.03	-0.18	0.02
11.68	III	0.07	0.02	0.12	0.03
11.39	III	0.86	0.05	1.12	0.04
11.29	III	-0.01	0.03	-0.22	0.01
11.09	III	-0.30	0.12	-0.38	0.03
10.89	II	0.02	0.05	-0.15	0.02
10.79	II	-0.06	0.03	-0.19	0.04
10.71	II	-0.03	0.05	-0.15	0.01
10.63	II	-0.23	0.03	-0.42	0.03
10.55	II	-0.34	0.01	-0.61	0.03
10.47	II	-0.21	0.03	-0.34	0.02
10.38	II	-0.15	0.02	-0.24	0.06
10.32	II	-0.23	0.04	-0.45	0.03
10.22	II	-0.10	0.03	-0.39	0.05
10.07	II	-0.07	0.05		
9.91	II	-0.08	0.02	-0.17	0.02
9.77	II	-0.10	0.01	-0.25	0.05
9.61	II	-0.09	0.04	-0.24	0.05
9.46	II	-0.01	0.03	-0.10	0.05
9.32	II	0.02	0.01	-0.08	0.04
9.16	II	0.00	0.00	-0.10	0.04
9.02	II	-0.04	0.05	-0.22	0.04
8.86	II	-0.02	0.05	-0.23	0.05
8.71	II	-0.06	0.03		
8.51	II	-0.02	0.03	0.18	0.03
8.3	II	-0.22	0.04	-0.18	0.03
8.1	II	-0.16	0.01	-0.02	0.07
7.91	II	-0.05	0.03	0.05	0.03
7.68	II	-0.26	0.03	-0.41	0.05
7.47	II	-0.15	0.04	-0.19	0.03
7.27	II	-0.09	0.03	-0.10	0.03
7.07	II	-0.08	0.01	-0.08	0.03
6.9	II	-0.13	0.04	-0.19	0.04

6.61	II	-0.07	0.02	-0.15	0.02
6.02	II	0.00	0.01	-0.07	0.02
5.72	I	0.05	0.01	0.01	0.06
5.42	I	0.00	0.03	0.01	0.01
5.12	I	-0.08	0.03	-0.04	0.03
4.82	I	-0.03	0.01	0.05	0.02
4.48	I	-0.15	0.01	0.12	0.02
4.18	I			-0.09	0.03
3.85	I	-0.02	0.02	-0.12	0.08
3.55	I	0.01	0.05	0.02	0.01
3.25	I	0.03	0.01		
2.95	I	0.02	0.05	-0.10	0.03
2.6	I	0.05	0.03	0.02	0.04
2.15	I	-0.12	0.03	-0.28	0.03
1.7	I	0.10	0.04		
1.38	I	0.18	0.03		
1.16	I	0.09	0.02	-0.09	0.08
0.74	I	0.02	0.03	-0.05	0.01
0.54	I	0.27	0.03		
0.41	I	0.15	0.04		

Table S2. Fe speciation, total Fe isotope ($\delta^{56}Fe_T$) and trace element (Mo, U, V) data for the Dob's Linn section, UK. *Fe speciation and trace element data for the samples at 11.45 m and 12.75 m are from ref (*24*).

Height (m)	Interval	Fe_{py} (%)	Fe_{carb} (%)	Fe_{mag} (%)	Fe_{ox} (%)	Fe_T (%)	Al (%)	Mo (ppm)	U (ppm)	V (ppm)	$\delta^{56}Fe_T$ (‰)	2SD (‰)
12.75*	III	2.18	0.74	0.06	0.06	3.99	6.05	17.69	8.59	98.10	0.12	0.03
11.45*	III	1.39	0.85	0.08	0.06	4.66	6.65	15.79	7.36	101.63	0.14	0.05
10.5	III	1.90	0.89	0.06	0.28	3.95	6.40	17.70	6.74	103.14	0.18	0.02
9.01	III	2.45	1.65	0.06	0.34	5.84	7.28	29.64	9.04	135.31	0.15	0.01
8	III	0.24	0.31	0.10	0.22	2.18	8.95	9.02	13.61	399.35	0.34	0.02
7.27	III	0.62	0.86	0.01	0.23	2.57	7.32	31.18	12.84	162.75	0.24	0.04
6.87	III	0.01	0.18	0.18	0.54	3.44	8.98	0.35	8.55	240.96	0.12	0.03
6.62	III	0.02	0.25	0.27	2.26	4.98	8.38	10.53	7.51	135.33	0.07	0.03
6.1	III	0.12	0.35	0.13	0.83	4.07	8.09	1.62	2.82	124.43	0.11	0.02
5.7	II	0.06	0.77	0.11	0.18	3.33	6.43	0.32	2.20	99.91	0.11	0.01
5.2	II	0.03	0.49	0.12	0.24	3.62	7.33	0.54	2.16	105.79	0.15	0.02
4.68	II	0.01	0.27	0.19	0.37	4.48	8.80	0.15	1.68	110.65	0.27	0.01
4.32	II	0.01	0.25	0.24	0.37	4.75	9.46	0.15	1.68	117.40	0.27	0.01
4.02	II	0.02	0.32	0.23	0.97	4.81	10.00	3.27	3.35	130.27	0.19	0.02
3.62	II	1.37	0.59	0.19	0.50	5.22	9.04	17.53	10.93	156.76	0.10	0.04
3.35	II	0.01	0.69	0.77	0.41	6.53	9.51	0.31	1.75	124.06		
2.94	I	0.04	0.26	0.09	0.34	1.66	8.94	21.30	11.36	198.74	0.41	0.03
2.67	I	0.14	0.30	0.15	0.38	3.98	8.73	1.89	2.08	116.12	0.17	0.04
2.45	I	0.06	0.07	0.09	0.57	1.95	8.46	10.68	7.89	142.43	0.32	0.02
2.1	I	0.11	0.22	0.17	0.44	4.76	9.27	1.19	2.29	133.21	0.12	0.04
1.78	I	0.01	0.24	0.22	0.26	5.01	9.73	0.18	1.57	105.89	0.17	0.03
1.25	I	0.00	0.24	0.32	0.36	4.61	8.97	0.20	1.71	112.09	0.18	0.02
0.74	I	0.01	0.23	0.25	0.43	4.66	9.31	0.64	2.63	148.30	0.20	0.01

Table S3. Organic carbon (C_{org}) contents, total P contents, P phase partitioning data and total P/Al ratios for the Dob's Linn section, UK. *C_{org} and P contents data for the samples at 11.45 m and 12.75 m are from ref (*24*).

Height/m	Interval	C_{org} (%)	P_{total} (ppm)	P_{auth} (ppm)	P_{cryst} (ppm)	P_{org} (ppm)	P_{mag} (ppm)	P_{Fe} (ppm)	P/Al (ppm/wt%)	Molar C_{org}/P_{org}	Molar C_{org}/P_{reac}
12.75	III	1.4*	247.9*	5.4	20.4	12.0	4.0	76.7	40.9	2934.5	359.3
11.45	III	1.2*	267.9*	48.9	34.0	10.7	5.6	49.3	40.3	2885.0	269.6
10.5	III	1.2	286.5	43.1	40.9	17.1	5.5	123.3	44.8	1825.9	165.2
9.01	III	1.3	210.6	5.8	12.5	12.5	2.8	124.9	28.9	2761.9	235.9
8	III	1.6	214.8	14.9	14.9	10.1	4.1	84.4	24.0	4157.9	370.9
7.27	III	1.7	128.0	3.1	10.4	42.9	0.6	34.3	17.5	1038.5	550.9
6.87	III	1.5	331.1	25.8	24.9	31.3	5.4	182.5	36.9	1271.0	162.4
6.62	III	0.8	672.4	41.2	34.3	10.8	11.0	480.8	80.3	1827.7	36.3
6.1	III	0.1	832.4	371.0	25.9	36.9	13.0	10.3	102.9	88.6	7.6
5.7	II	0.1	1079.4	87.1	422.9	35.0	41.7	1.3	167.8	78.0	16.6
5.2	II	0.1	628.3	200.6	324.8	4.2	15.3	1.3	85.7	599.8	11.5
4.68	II	0.1	428.5	211.3	135.2	6.1	10.9	1.3	48.7	453.3	12.1
4.32	II	0.2	360.5	165.9	70.9	6.2	8.8	22.1	38.1	891.0	27.3
4.02	II	0.1	401.3	33.2	22.3	7.2	5.0	248.0	40.1	280.1	6.9
3.62	II	0.1	625.2	201.8	99.4	42.2	11.0	211.3	69.2	63.8	5.8
3.35	II	0.1	367.8	163.1	64.7	11.5	6.8	26.0	38.7	173.4	9.6
2.94	I	1.8	207.6	20.9	14.8	65.4	5.3	67.4	23.2	715.4	294.2
2.67	I	0.1	423.8	98.3	40.7	23.6	5.5	190.7	48.5	97.0	7.2
2.45	I	1.8	360.5	14.2	16.8	64.5	3.7	190.7	42.6	728.3	172.1
2.1	I	0.1	365.3	126.9	54.5	5.6	5.9	80.4	39.4	487.8	12.4
1.78	I	0.1	378.3	154.1	52.5	11.1	7.7	61.7	38.9	288.0	13.6
1.25	I	0.1	328.5	88.8	34.6	17.9	4.4	117.7	36.6	153.5	12.0
0.74	I	0.1	391.8	127.5	35.2	6.0	5.8	111.8	42.1	295.6	7.0

REFERENCES

1. G. M. Narbonne, The Ediacara biota: Neoproterozoic origin of animals and their ecosystems. *Annu. Rev. Earth Planet. Sci.* **33**, 421–442 (2005).

2. D. E. Canfield, S. W. Poulton, G. M. Narbonne, Late-Neoproterozoic deep-ocean oxygenation and the rise of animal life. *Science* **315**, 92–95 (2007).

3. D. B. Cole, D. B. Mills, D. H. Erwin, E. A. Sperling, S. M. Porter, C. T. Reinhard, N. J. Planavsky, On the co-evolution of surface oxygen levels and animals. *Geobiology* **18**, 260–281 (2020).

4. E. R. Haxen, N. H. Schovsbo, A. T. Nielsen, S. Richoz, D. K. Loydell, N. R. Posth, D. E. Canfield, E. U. Hammarlund, "Hypoxic" Silurian oceans suggest early animals thrived in a low-O_2 world. *Earth Planet. Sci. Lett.* **622**, 118416 (2023).

5. R. D. Alexander, A. Y. Zhuravlev, F. T. Bowyer, L. Pichevin, S. W. Poulton, A. Kouchinsky, R. Wood, Low oxygen but dynamic marine redox conditions permitted the Cambrian Radiation. *Sci. Adv.* **11**, eads2846 (2025).

6. K. Feng, F. Bowyer, A. Curtis, S. W. Poulton, L. Pichevin, R. Wood, Persistent dysoxia in very shallow seas across the late Cambrian SPICE event, Durness Group, UK. *Geology* **53**, 642–646 (2025).

7. R. G. Stockey, D. B. Cole, U. C. Farrell, H. Agić, T. H. Boag, J. J. Brocks, D. E. Canfield, M. Cheng, P. W. Crockford, H. Cui, T. W. Dahl, L. Del Mouro, K. Dewing, S. Q. Dornbos, J. F. Emmings, R. R. Gaines, T. M. Gibson, B. C. Gill, G. J. Gilleaudeau, K. Goldberg, R. Guilbaud, G. Halverson, E. U. Hammarlund, K. Hantsoo, M. A. Henderson, C. M. Henderson, M. S. W. Hodgskiss, A. J. M. Jarrett, D. T. Johnston, P. Kabanov, J. Kimmig, A. H. Knoll, M. Kunzmann, M. A. LeRoy, C. Li, D. K. Loydell, F. A. Macdonald, J. M. Magnall, N. T. Mills, L. M. Och, B. O'Connell, A. Pagès, S. E. Peters, S. M. Porter, S. W. Poulton, S. R. Ritzer, A. D. Rooney, S. Schoepfer, E. F. Smith, J. V. Strauss, G. J. Uhlein, T. White, R. A. Wood, C. R. Woltz, I. Yurchenko, N. J. Planavsky, E. A. Sperling, Sustained increases in atmospheric oxygen and marine productivity in the Neoproterozoic and Palaeozoic eras. *Nat. Geosci.* **17**, 667–674 (2024).

8. E. A. Sperling, M. J. Melchin, T. Fraser, R. G. Stockey, U. C. Farrell, L. Bhajan, T. N. Brunoir, D. B. Cole, B. C. Gill, A. Lenz, D. K. Loydell, J. Malinowski, A. J. Miller, S. Plaza-Torres, B. Bock, A. D. Rooney, S. A. Tecklenburg, J. M. Vogel, N. J. Planavsky, J. V. Strauss, A long-term record of early to mid-Paleozoic marine redox change. *Sci. Adv.* **7**, eabf4382 (2021).

9. T. W. Dahl, E. U. Hammarlund, A. D. Anbar, D. P. G. Bond, B. C. Gill, G. W. Gordon, A. H. Knoll, A. T. Nielsen, N. H. Schovsbo, D. E. Canfield, Devonian rise in atmospheric oxygen correlated to the radiations of terrestrial plants and large predatory fish. *Proc. Natl. Acad. Sci. U.S.A.* **107**, 17911–17915 (2010).

10. C. Zou, Z. Qiu, S. W. Poulton, D. Dong, H. Wang, D. Chen, B. Lu, Z. Shi, H. Tao, Ocean euxinia and climate change "double whammy" drove the Late Ordovician mass extinction. *Geology* **46**, 535–538 (2018).

11. N. P. Kozik, S. A. Young, S. M. Newby, M. Liu, D. Chen, E. U. Hammarlund, D. P. G. Bond, T. R. Them, J. D. Owens, Rapid marine oxygen variability: Driver of the Late Ordovician mass extinction. *Sci. Adv.* **8**, eabn8345 (2022).

12. T. W. Dahl, E. U. Hammarlund, C. M. Ø. Rasmussen, D. P. G. Bond, D. E. Canfield, Sulfidic anoxia in the oceans during the Late Ordovician mass extinctions—Insights from molybdenum and uranium isotopic global redox proxies. *Earth Sci. Rev.* **220**, 103748 (2021).

13. E. U. Hammarlund, T. W. Dahl, D. A. T. Harper, D. P. G. Bond, A. T. Nielsen, C. J. Bjerrum, N. H. Schovsbo, H. P. Schönlaub, J. A. Zalasiewicz, D. E. Canfield, A sulfidic driver for the end-Ordovician mass extinction. *Earth Planet. Sci. Lett.* **331–332**, 128–139 (2012).

14. C. M. Ø. Rasmussen, T. R. A. Vandenbroucke, D. Nogues-Bravo, S. Finnegan, Was the Late Ordovician mass extinction truly exceptional? *Trends Ecol. Evol.* **38**, 812–821 (2023).

15. D. Jablonski, Extinctions: A paleontological perspective. *Science* **253**, 754–757 (1991).

16. P. Storch, C. E. Mitchell, S. C. Finney, M. J. Melchin, Uppermost Ordovician (upper Katian-Hirnantian) graptolites of north-central Nevada, U.S.A. *Bull. Geosci.* **86**, 301–386 (2011).

17. M. J. Melchin, C. E. Mitchell, A. Naczk-Cameron, J. X. Fan, J. Loxton, Phylogeny and adaptive radiation of the Neograptina (Graptoloida) during the Hirnantian mass extinction and Silurian recovery. *Proc. Yorks. Geol. Soc.* **58**, 281–309 (2011).

18. H. D. Sheets, C. E. Mitchell, M. J. Melchin, J. Loxton, P. Štorch, K. L. Carlucci, A. D. Hawkins, Graptolite community responses to global climate change and the Late Ordovician mass extinction. *Proc. Natl. Acad. Sci. U.S.A.* **113**, 8380–8385 (2016).

19. D. W. Bapst, P. C. Bullock, M. J. Melchin, H. D. Sheets, C. E. Mitchell, Graptoloid diversity and disparity became decoupled during the Ordovician mass extinction. *Proc. Natl. Acad. Sci. U.S.A.* **109**, 3428–3433 (2012).

20. J. Fan, X. Chen, Preliminary report on the Late Ordovician graptolite extinction in the Yangtze region. *Palaeogeogr. Palaeoclimatol. Palaeoecol.* **245**, 82–94 (2007).

21. X. Chen, M. J. Melchin, H. D. Sheets, C. E. Mitchell, J. X. Fan, Patterns and processes of latest Ordovician graptolite extinction and recovery based on data from South China. *J. Paleontol.* **79**, 842–861 (2005).

22. S. C. Finney, W. B. N. Berry, J. D. Cooper, The influence of denitrifying seawater on graptolite extinction and diversification during the Hirnantian (latest Ordovician) mass extinction event. *Lethaia* **40**, 281–291 (2007).

23. Z. Qiu, C. Zou, B. J. W. Mills, Y. Xiong, H. Tao, B. Lu, H. Liu, W. Xiao, S. W. Poulton, A nutrient control on expanded anoxia and global cooling during the Late Ordovician mass extinction. *Commun. Earth Environ.* **3**, 82 (2022).

24. A. Sánchez-Roda, P. B. Wignall, Y. Xiong, S. W. Poulton, Redox changes in the Iapetus Ocean during the Late Ordovician extinction crises. *J. Geol. Soc. London* **181**, jgs2023-195 (2024).

25. T. Tyrrell, The relative influences of nitrogen and phosphorus on oceanic primary production. *Nature* **400**, 525–531 (1999).

26. S. C. Finney, W. B. N. Berry, New perspectives on graptolite distributions and their use as indicators of platform margin dynamics. *Geology* **25**, 919–922 (1997).

27. C. P. Slomp, E. H. Epping, W. Helder, W. V. Raaphorst, A key role for iron-bound phosphorus in authigenic apatite formation in North Atlantic continental platform sediments. *J. Mar. Res.* **54**, 1179–1205 (1996).

28. C. P. Slomp, W. van Raaphorst, Phosphate adsorption in oxidized marine sediments. *Chem. Geol.* **107,** 477–480 (1993).

29. I. Tsandev, D. C. Reed, C. P. Slomp, Phosphorus diagenesis in deep-sea sediments: Sensitivity to water column conditions and global scale implications. *Chem. Geol.* **330–331,** 127–139 (2012).

30. E. D. Ingall, R. M. Bustin, P. Van Cappellen, Influence of water column anoxia on the burial and preservation of carbon and phosphorus in marine shales. *Geochim. Cosmochim. Acta* **57**, 303–316 (1993).

31. K. C. Ruttenberg, R. A. Berner, Authigenic apatite formation and burial in sediments from non-upwelling, continental margin environments. *Geochim. Cosmochim. Acta* **57**, 991–1007 (1993).

32. R. Guilbaud, S. W. Poulton, J. Thompson, K. F. Husband, M. Zhu, Y. Zhou, G. A. Shields, T. M. Lenton, Phosphorus-limited conditions in the early Neoproterozoic ocean maintained low levels of atmospheric oxygen. *Nat. Geosci.* **13**, 296–301 (2020).

33. E. Ingall, R. Jahnke, Evidence for enhanced phosphorus regeneration from marine sediments overlain by oxygen depleted waters. *Geochim. Cosmochim. Acta* **58**, 2571–2575 (1994).

34. S. W. Poulton, M. D. Krom, R. Raiswell, A revised scheme for the reactivity of iron (oxyhydr)oxide minerals towards dissolved sulfide. *Geochim. Cosmochim. Acta* **68**, 3703–3715 (2004).

35. Y. Xiong, R. Guilbaud, C. L. Peacock, M. D. Krom, S. W. Poulton, Phosphorus controls on the formation of vivianite versus green rust under anoxic conditions. *Geochim. Cosmochim. Acta* **351**, 139–151 (2023).

36. N. M. Papadomanolaki, W. K. Lenstra, M. Wolthers, C. P. Slomp, Enhanced phosphorus recycling during past oceanic anoxia amplified by low rates of apatite authigenesis. *Sci. Adv.* **8**, eabn2370 (2022).

37. S. W. Poulton, D. E. Canfield, Ferruginous conditions: A dominant feature of the ocean through Earth's history. *Elements* **7**, 107–112 (2011).

38. S. W. Poulton, *The Iron Speciation Paleoredox Proxy* (Cambridge Univ. Press, 2021).

39. S. Li, P. B. Wignall, S. W. Poulton, Co-application of rhenium, vanadium, uranium and molybdenum as paleo-redox proxies: Insight from modern and ancient environments. *Chem. Geol.* **674**, 122565 (2025).

40. B. L. Beard, C. M. Johnson, J. L. Skulan, K. H. Nealson, L. Cox, H. Sun, Application of Fe isotopes to tracing the geochemical and biological cycling of Fe. *Chem. Geol.* **195**, 87–117 (2003).

41. M. Kunzmann, T. M. Gibson, G. P. Halverson, M. S. W. Hodgskiss, T. H. Bui, D. A. Carozza, E. A. Sperling, A. Poirier, G. M. Cox, B. A. Wing, Iron isotope biogeochemistry of Neoproterozoic marine shales. *Geochim. Cosmochim. Acta* **209**, 85–105 (2017).

42. J. Thompson, S. W. Poulton, R. Guilbaud, K. A. Doyle, S. Reid, M. D. Krom, Development of a modified SEDEX phosphorus speciation method for ancient rocks and modern iron-rich sediments. *Chem. Geol.* **524**, 383–393 (2019).

43. X. Chen, J. Rong, J. Fan, R. Zhan, E. M. Charles, A. T. H. David, J. M. Michael, C. F. Stan, X. Wang, The Global Boundary Stratotype Section and Point (GSSP) for the base of the Hirnantian Stage (the uppermost of the Ordovician System). *Episodes* **29**, 183–196 (2006).

44. S. Williams, Dob's Linn-the Ordovician-Silurian boundary stratotype. *Bull. Br. Mus. (Nat. Hist.) Geol.* **43**, 17–30 (1988).

45. R. Raiswell, D. E. Canfield, Sources of iron for pyrite formation in marine sediments. *Am. J. Sci.* **298**, 219–245 (1998).

46. S. W. Poulton, D. E. Canfield, Development of a sequential extraction procedure for iron: Implications for iron partitioning in continentally derived particulates. *Chem. Geol.* **214**, 209–221 (2005).

47. M. Mansor, M. S. Fantle, A novel framework for interpreting pyrite-based Fe isotope records of the past. *Geochim. Cosmochim. Acta* **253**, 39–62 (2019).

48. R. Guilbaud, I. B. Butler, R. M. Ellam, Abiotic pyrite formation produces a large Fe isotope fractionation. *Science* **332**, 1548–1551 (2011).

49. J. Dupeyron, M. N. Decraene, J. Marin-Carbonne, V. Busigny, Formation pathways of Precambrian sedimentary pyrite: Insights from in situ Fe isotopes. *Earth Planet. Sci. Lett.* **609**, 118070 (2023).

50. S. M. McLennan, Relationships between the trace element composition of sedimentary rocks and upper continental crust. *Geochem. Geophys. Geosyst.* **2**, 1021 (2001).

51. D. Yan, D. Chen, Q. Wang, J. Wang, Predominance of stratified anoxic Yangtze Sea interrupted by short-term oxygenation during the Ordo-Silurian transition. *Chem. Geol.* **291**, 69–78 (2012).

52. T. J. Algeo, N. Tribovillard, Environmental analysis of paleoceanographic systems based on molybdenum–uranium covariation. *Chem. Geol.* **268**, 211–225 (2009).

53. G. R. Helz, Dissolved molybdenum asymptotes in sulfidic waters. *Geochem. Perspect. Lett.* **19**, 23–26 (2021).

54. S. R. Emerson, S. S. Huested, Ocean anoxia and the concentrations of molybdenum and vanadium in seawater. *Mar. Chem.* **34**, 177–196 (1991).

55. Y. Song, F. T. Bowyer, B. J. W. Mills, A. S. Merdith, P. B. Wignall, J. Peakall, S. Zhang, X. Wang, H. Wang, D. E. Canfield, G. A. Shields, S. W. Poulton, Dynamic redox and nutrient cycling response to climate forcing in the Mesoproterozoic ocean. *Nat. Commun.* **14**, 6640 (2023).

56. C. Zou, Z. Qiu, H. Wei, D. Dong, B. Lu, Euxinia caused the Late Ordovician extinction: Evidence from pyrite morphology and pyritic sulfur isotopic composition in the Yangtze area, South China. *Palaeogeogr. Palaeoclimatol. Palaeoecol.* **511**, 1–11 (2018).

57. P. Wignall, R. Newton, Pyrite framboid diameter as a measure of oxygen deficiency in ancient mudrocks. *Am. J. Sci.* **298**, 537–552 (1998).

58. A. McAnena, S. Severmann, R. Guilbaud, S. W. Poulton, Iron isotope fractionation during sulfide-promoted reductive dissolution of iron (oxyhydr)oxide minerals. *Geochim. Cosmochim. Acta* **369**, 17–34 (2024).

59. S. Severmann, C. M. Johnson, B. L. Beard, J. McManus, The effect of early diagenesis on the Fe isotope compositions of porewaters and authigenic minerals in continental margin sediments. *Geochim. Cosmochim. Acta* **70**, 2006–2022 (2006).

60. A. Grengs, G. Ledesma, Y. Xiong, S. Katsev, S. W. Poulton, E. D. Swanner, C. Wittkop, Direct precipitation of siderite in ferruginous environments. *Geochem. Perspect. Lett.* **30**, 1–6 (2024).

61. R. A. Wiesli, B. L. Beard, C. M. Johnson, Experimental determination of Fe isotope fractionation between aqueous Fe(II), siderite and "green rust" in abiotic systems. *Chem. Geol.* **211**, 343–362 (2004).

62. C. M. Johnson, E. E. Roden, S. A. Welch, B. L. Beard, Experimental constraints on Fe isotope fractionation during magnetite and Fe carbonate formation coupled to dissimilatory hydrous ferric oxide reduction. *Geochim. Cosmochim. Acta* **69**, 963–993 (2005).

63. G. A. Icopini, A. D. Anbar, S. S. Ruebush, M. Tien, S. L. Brantley, Iron isotope fractionation during microbial reduction of iron: The importance of adsorption. *Geology* **32**, 205–208 (2004).

64. L. Wu, B. L. Beard, E. E. Roden, C. M. Johnson, Stable iron isotope fractionation between aqueous Fe(II) and hydrous ferric oxide. *Environ. Sci. Technol.* **45**, 1847–1852 (2011).

65. A. J. Frierdich, B. L. Beard, M. M. Scherer, C. M. Johnson, Determination of the Fe(II)aq–magnetite equilibrium iron isotope fractionation factor using the three-isotope method and a multi-direction approach to equilibrium. *Earth Planet. Sci. Lett.* **391**, 77–86 (2014).

66. S. W. Poulton, R. Raiswell, The low-temperature geochemical cycle of iron: From continental fluxes to marine sediment deposition. *Am. J. Sci.* **302**, 774–805 (2002).

67. A. Tagliabue, L. Bopp, J.-C. Dutay, A. R. Bowie, F. Chever, P. Jean-Baptiste, E. Bucciarelli, D. Lannuzel, T. Remenyi, G. Sarthou, O. Aumont, M. Gehlen, C. Jeandel, Hydrothermal contribution to the oceanic dissolved iron inventory. *Nat. Geosci.* **3**, 252–256 (2010).

68. W. B. Homoky, S. G. John, T. M. Conway, R. A. Mills, Distinct iron isotopic signatures and supply from marine sediment dissolution. *Nat. Commun.* **4**, 2143 (2013).

69. S. Severmann, T. W. Lyons, A. Anbar, J. McManus, G. Gordon, Modern iron isotope perspective on the benthic iron shuttle and the redox evolution of ancient oceans. *Geology* **36**, 487–490 (2008).

70. F. Scholz, S. Severmann, J. McManus, C. Hensen, Beyond the Black Sea paradigm: The sedimentary fingerprint of an open-marine iron shuttle. *Geochim. Cosmochim. Acta* **127**, 368–380 (2014).

71. L. Nana Yobo, H. M. Williams, A. D. Brandon, C. Holmden, K. V. Lau, S. C. Bergman, J. S. Eldrett, D. Minisini, Iron Isotopes reveal volcanogenic input during Oceanic Anoxic Event 2 (OAE 2 ~ 94 Ma). *Geochim. Cosmochim. Acta* **389**, 157–167 (2025).

72. J. D. Owens, T. W. Lyons, X. Li, K. G. Macleod, G. Gordon, M. M. M. Kuypers, A. Anbar, W. Kuhnt, S. Severmann, Iron isotope and trace metal records of iron cycling in the proto-North Atlantic during the Cenomanian-Turonian oceanic anoxic event (OAE-2). *Paleoceanography* **27**, PA3223 (2012).

73. Y. Xiong, R. Guilbaud, C. L. Peacock, R. P. Cox, D. E. Canfield, M. D. Krom, S. W. Poulton, Phosphorus cycling in Lake Cadagno, Switzerland: A low sulfate euxinic ocean analogue. *Geochim. Cosmochim. Acta* **251**, 116 – 135 (2019).

74. W. B. Berry, The Ordovician-Silurian boundary: New data, new concerns. *Lethaia* **20**, 209–216 (1987).

75. W. B. Berry, P. Wilde, M. S. Quinby-Hunt, The oceanic non-sulfidic oxygen minimum zone: A habitat for graptolites. *Bull. Geol. Soc. Den.* **35**, 103–114 (1986).

76. M. J. Melchin, C. E. Mitchell, C. Barnes, S. Williams, Late Ordovician extinction in the Graptoloidea. *Adv. Ordovician Geol.* **90**, 143–156 (1991).

77. J. Longman, B. J. W. Mills, H. R. Manners, T. M. Gernon, M. R. Palmer, Late Ordovician climate change and extinctions driven by elevated volcanic nutrient supply. *Nat. Geosci.* **14**, 924–929 (2021).

78. L. J. Alcott, A. J. Krause, E. U. Hammarlund, C. J. Bjerrum, F. Scholz, Y. Xiong, A. J. Hobson, L. Neve, B. J. W. Mills, C. März, B. Schnetger, A. Bekker, S. W. Poulton, Development of iron speciation reference materials for palaeoredox analysis. *Geostand. Geoanal. Res.* **44**, 581–591 (2020).

79. M. A. Huerta-Diaz, J. W. Morse, A quantitative method for determination of trace metal concentrations in sedimentary pyrite. *Mar. Chem.* **29**, 119–144 (1990).

80. S. Henkel, S. Kasten, S. W. Poulton, M. Staubwasser, Determination of the stable iron isotopic composition of sequentially leached iron phases in marine sediments. *Chem. Geol.* **421**, 93–102 (2016).

81. P. B. H. Oonk, H. Tsikos, P. R. D. Mason, S. Henkel, M. Staubwasser, L. Fryer, S. W. Poulton, H. M. Williams, Fraction-specific controls on the trace element distribution in iron formations: Implications for trace metal stable isotope proxies. *Chem. Geol.* **474**, 17–32 (2017).

82. D. E. Canfield, R. Raiswell, J. T. Westrich, C. M. Reaves, R. A. Berner, The use of chromium reduction in the analysis of reduced inorganic sulfur in sediments and shales. *Chem. Geol.* **54**, 149–155 (1986).

83. F. Koroleff, "Determination of phosphorus" in *Methods of Seawater Analysis* (Verlag Chemie, ed. 2, 1983), pp. 125–131.

84. R. F. Anderson, M. Q. Fleisher, A. P. LeHuray, Concentration, oxidation state, and particulate flux of uranium in the Black Sea. *Geochim. Cosmochim. Acta* **53**, 2215–2224 (1989).

85. N. Tribovillard, T. J. Algeo, T. Lyons, A. Riboulleau, Trace metals as paleoredox and paleoproductivity proxies: An update. *Chem. Geol.* **232**, 12–32 (2006).

86. M. Melchin, S. Williams, "A restudy of the akidograptine graptolites from Dob's Linn and a proposed redefined zonation of the Silurian stratotype," in *Palaeontology Down-Under 2000* (Geological Society of Australia, 2000), p. 63.

87. A. Pohl, Z. Lu, W. Lu, R. G. Stockey, M. Elrick, M. Li, A. Desrochers, Y. Shen, R. He, S. Finnegan, A. Ridgwell, Vertical decoupling in Late Ordovician anoxia due to reorganization of ocean circulation. *Nat. Geosci.* **14**, 868 – 873 (2021).

88. L. Zhou, T. J. Algeo, J. Shen, Z. Hu, H. Gong, S. Xie, J. Huang, S. Gao, Changes in marine productivity and redox conditions during the Late Ordovician Hirnantian glaciation. *Palaeogeogr. Palaeoclimatol. Palaeoecol.* **420**, 223 – 234 (2015).

89. R. Bartlett, M. Elrick, J. R. Wheeley, V. Polyak, A. Desrochers, Y. Asmerom, Abrupt global-ocean anoxia during the Late Ordovician – early Silurian detected using uranium isotopes of marine carbonates. *Proc. Natl. Acad. Sci. U.S.A.* **115**, 5896 – 5901 (2018).

90. M. Liu, D. Chen, L. Jiang, R. G. Stockey, D. Aseal, B. Zhang, K. Liu, X. Yang, D. Yan, N. J. Planavsky, Oceanic anoxia and extinction in the latest Ordovician. *Earth Planet. Sci. Lett.* **588**, 117553 (2022).

91. S. Lin, L. Wang, W. Xiao, G. Xing, Z. Niu, X. Zhao, C. Yin, S. Zhang, H. Liu, The early Paleozoic Wuyi–Yunkai orogeny in South China: A collisional orogeny with a major lag in time between onset of collision and peak metamorphism in subducted continental crust. *Geol. Soc. Spe. Publ.* **542**, 619–641 (2024).

92. R. Zhan, J. Jin, J. Liu, P. Corcoran, X. Luan, X. Wei, Meganodular limestone of the Pagoda Formation: A time-specific carbonate facies in the Upper Ordovician of South China. *Palaeogeogr. Palaeoclimatol. Palaeoecol.* **448**, 349–362 (2016).

93. X. Chen, J. Fan, Q. Chen, L. Tang, X. Hou, Toward a stepwise Kwangsian Orogeny. *Sci. China Earth Sci.* **57**, 379–387 (2014).

94. J. Rong, D. A. T. Harper, B. Huang, R. Li, X. Zhang, D. Chen, The latest Ordovician Hirnantian brachiopod faunas: New global insights. *Earth Sci. Rev.* **208**, 103280 (2020).

95. Z. Qiu, B. Liu, B. Lu, Z. Shi, Z. Li, Mineralogical and petrographic characteristics of the Ordovician-Silurian Wufeng-Longmaxi Shale in the Sichuan Basin and implications for depositional conditions and diagenesis of black shales. *Mar. Pet. Geol.* **135**, 105428 (2022).

96. S. H. Williams, The Ordovician-Silurian boundary graptolite fauna of Dob's Linn, southern Scotland. *Palaeontology* **26**, 605–639 (1983).

97. J. Rong, M. Melchin, S. H. Williams, T. N. Koren, J. Verniers, Report of the restudy of the defined global stratotype of the base of the Silurian System. *Episodes* **31**, 315–318 (2008).

98. H. A. Armstrong, A. L. Coe, Deep-sea sediments record the geophysiology of the late Ordovician glaciation. *J. Geol. Soc. London* **154**, 929–934 (1997).

99. H. K. Garza, E. J. Catlos, K. R. Chamberlain, S. E. Suarez, M. E. Brookfield, D. F. Stockli, R. A. Batchelor, How old is the Ordovician–Silurian boundary at Dob's Linn, Scotland? Integrating LA-ICP-MS and CA-ID-TIMS U-Pb zircon dates. *Geol. Mag.* **160**, 1775–1789 (2023).

100. J. Zalasiewicz, Graptolites as constraints on models of sedimentation across Iapetus: A review. *Proc. Geol. Assoc.* **112**, 237–251 (2001).

101. R. T. Wilkin, H. L. Barnes, S. L. Brantley, The size distribution of framboidal pyrite in modern sediments: An indicator of redox conditions. *Geochim. Cosmochim. Acta* **60**, 3897–3912 (1996).

102. R. T. Wilkin, H. L. Barnes, Formation processes of framboidal pyrite. *Geochim. Cosmochim. Acta* **61**, 323–339 (1997).

103. I. B. Butler, C. Archer, D. Vance, A. Oldroyd, D. Rickard, Fe isotope fractionation on FeS formation in ambient aqueous solution. *Earth Planet. Sci. Lett.* **236**, 430–442 (2005).

104. R. Guilbaud, I. B. Butler, R. M. Ellam, D. Rickard, A. Oldroyd, Experimental determination of the equilibrium Fe isotope fractionation between and FeSm (mackinawite) at 25 and 2°C. *Geochim. Cosmochim. Acta* **75**, 2721–2734 (2011).

105. L. Wu, G. Druschel, A. Findlay, B. L. Beard, C. M. Johnson, Experimental determination of iron isotope fractionations amongFeaq2+–FeSaq–Mackinawite at low temperatures: Implications for the rock record. *Geochim. Cosmochim. Acta* **89**, 46–61 (2012).

106. J. M. Rolison, C. H. Stirling, R. Middag, M. Gault-Ringold, E. George, M. J. A. Rijkenberg, Iron isotope fractionation during pyrite formation in a sulfidic Precambrian ocean analogue. *Earth Planet. Sci. Lett.* **488**, 1–13 (2018).

107. D. D. Syverson, D. M. Borrok, W. E. Seyfried, Experimental determination of equilibrium Fe isotopic fractionation between pyrite and dissolved Fe under hydrothermal conditions. *Geochim. Cosmochim. Acta* **122**, 170–183 (2013).
